# Crystal Structure Determination of 4-[(Di-p-tolyl-amino)-benzylidene]-(5-pyridin-4-yl-[1,3,4]thiadiazol-2-yl)-imine along with Selected Properties of Imine in Neutral and Protonated Form with Camforosulphonic Acid: Theoretical and Experimental Studies

**DOI:** 10.3390/ma14081952

**Published:** 2021-04-13

**Authors:** Agnieszka Dylong, Karolina Dysz, Krzysztof A. Bogdanowicz, Wojciech Przybył, Krzysztof A. Konieczny, Ilona Turowska-Tyrk, Andrzej Kaim, Agnieszka Iwan

**Affiliations:** 1 Military Institute of Engineer Technology, 136 Obornicka Str., 50-961 Wroclaw, Poland; dylong@witi.wroc.pl (A.D.); dysz@witi.wroc.pl (K.D.); bogdanowicz@witi.wroc.pl (K.A.B.); przybyl@witi.wroc.pl (W.P.); 2 Faculty of Chemistry, Wrocław University of Science and Technology, 27 Wybrzeże Wyspiańskiego, 50-370 Wroclaw, Poland; krzysztof.konieczny@pwr.edu.pl (K.A.K.); ilona.turowska-tyrk@pwr.edu.pl (I.T.-T.); 3 Faculty of Chemistry, University of Warsaw, 1 Pasteura Str., 02-093 Warsaw, Poland; akaim@chem.uw.edu.pl

**Keywords:** imines, azomethines, crystal structure, non-covalent interactions, camforosulphonic acid, thermographic camera, protonation

## Abstract

The crystal structure was determined for the first time for 4-[(di-p-tolyl-amino)benzylidene]-(5-pyridin-4-yl-[1,3,4]thiadiazol-2-yl)-imine (*trans*-PPL9) by X-ray diffraction. The imine crystallized in the monoclinic P2_1_/n space group with a = 18.9567(7) Å, b = 6.18597(17) Å, c = 22.5897(7) Å, and β = 114.009(4)°. Intermolecular interactions in the PPL9 crystal were only weak C−H⋯N hydrogen bonds investigated using the Hirshfeld surface. The electronic and geometric structure of the imine were investigated by the density functional theory and the time-dependent density-functional theory. The properties of the imine in neutral and protonated form with camforosulphonic acid (CSA) were investigated using cyclic voltammetry, UV–vis and ^1^H NMR spectroscopy. Theoretical and experimental studies showed that for the 1:1 molar ratio the protonation occured on nitrogen in pyridine in the PPL9 structure, as an effect of Brönsted acid–base interactions. Thermographic camera was used to defined defects in constructed simple devices with ITO/PPL9 (or PPL9:CSA)/Ag/ITO architecture. In conclusion, a thermally stable imine was synthesized in crystalline form and by CSA doping, a modification of absorption spectra together with reduction of overheating process was observed, suggesting its potential application in optoelectronics.

## 1. Introduction

Imines (azomethines, Schiff bases) are currently investigated as new compounds in three main streams: organic optoelectronic devices, as biological compounds and as admixtures for organic and organic-inorganic compositions for electrical applications [1,2,3,4,5,6,7]. It should be emphasized that imines are extensively studied group of materials due to tunability of their thermal, optical and electrical properties, adding adequate protonating agent [8,9,10,11]. In our previous research [12] we described that polyketanils with 3,8-diamino-6-phenylphenanthridine groups doped with CSA and methanesulfonic acid (MSA) change their photoluminescence properties differently, depending on used solvent. It was found that both CSA and MSA interacted mainly with the nitrogen in phenanthridine ring of the polyketanils. The impact of the chemical structure of Schiff bases containing triphenylamine (TPA) and pyridine groups on their optical and structural properties were also wide studied [13,14,15,16,17,18,19].

Unfortunately, many studies are devoted to the analysis of the properties of liquid crystal imines with an aromatic-aliphatic structure, whereas only few studies concerned the analysis of the properties of imines obtained in the form of a single crystal [20,21,22,23,24,25,26,27,28,29,30,31,32,33,34,35,36,37,38,39]. There is a little information on the new Schiff bases synthesized as a crystal and molecular interaction occurring in such structures: for example, Pickering et al. [40] investigated the significance of π–π, π–CH and N=CH interactions for the packing of cis, cis- and cis, trans-1,3,5-triaminocyclohexane with Schiff’s base. Lai et al. [41] investigated symmetrical imine with cyclohexane and pyridine moieties and their intermolecular interaction. Interesting work was done by Bolduc et al. [42]; authors obtained an azomethine derived from EDOT segments as single crystal via slow evaporation of the solvent and investigated its electrochromic properties (the imine exhibited reversible oxidation). Single crystal X-ray diffraction (SCXRD) confirmed that the imine crystal structure consists of two diaminothiophene units and one ethylenedioxythiophene group, and indicating also the *E* conformation of the heteroatom double bond. Sanchez et al. [43] described the synthesis of Schiff bases and their photochromic behavior as an effect of intermolecular hydrogen bond formation. It was demonstrated that only Schiff’s base derivatives of phenylglycinol showed photochromism in the solid state.

Some research was also dedicated to investigation of the influence of Lewis acids on selected properties of Schiff bases, also for molecules containing pyridine moieties [44,45,46]. Additionally, ligands based on the Schiff bases with pyridine are also investigated in coordination chemistry [47]. Moreover, Schiff bases with pyridine core are known antimicrobial, anti-inflammatory, anti-tubercular, analgesic, anti-tumor, antioxidant, anti-hypertensive, and anti-convulsive agents [48,49,50]. Another interesting compounds, next to pyridine, are 1,3,4-thiadazoles a vital class of heterocycles, due to their broad spectrum of biological activity [51]. 

From the application point of view, while designing molecular structure it has been noticed that the incorporation of bulky triphenylamine (TPA) into the chemical structure of the imine structure is likely to reduce the tendency to intermolecular aggregation, reducing the tendency to crystallize and improving the hole transporting ability of the material [52]. Therefore, the presence of triphenylamine in the structure conveys the hole conduction properties on new molecules, hence is often used as a building block for organic compounds. As an example of hole-transporting material for perovskite solar cells, can be given an imine with thiadiazole and TPA units for which perovskite solar cells exhibited the highest value of power conversion efficiency (PCE = 14.4%) without hysteresis effect and it is the best reported value for this class of materials [53]. Earlier, Petrus et al. [54] used other small-molecule azomethine with TPA units in perovskite solar cells with the PCE = 11%, the previous record. It should be also mentioned that the TPA is commonly used as a photoconductor in the XeroxR process in laser printers and photocopiers.

Herein, we present the crystal structure of 4-[(di-p-tolyl-amino)-benzylidene]-(5-pyridin-4-yl-[1,3,4]thiadiazol-2-yl)-imine synthesized in a single-step condensation method in solution followed by crystallization in cold hexane. To the best of our knowledge, it is the first example of unsymmetrical imine with pyridine, thiadiazole and triphenylamine building blocks in a monocrystal form. Theoretical and experimental methods were used to characterized imine in detail, to provide an insight into non-covalent interactions using Hirshfeld Surface Analysis. It should be highlighted that this contribution is one of few reports on non-covalent interactions studied by means of Hirshfeld surface analysis for imine with pyridine moieties [55,56]. Additionally, UV–vis and ^1^H NMR spectroscopy were used to describe the influence of the CSA doping level on selected optical and structural properties of the imine. It is the first report providing correlation between molecular structure and tendency to form crystalline structure of imine containing TPA unit and the influence of acidic dopant on imine’s optical properties. Additionally, electric conductivity, structural defects and morphology of the layers formed by CSA doped imine were studied by thermal imaging. 

The motivation for performing a structure optimization is to determine the ground state parameters of the studied system. It means that the energy of the system is the lowest. The optimized parameters (arrangement of the atoms, chemical bond lengths, angles, torsion angles...). Then, these parameters can be used in a variety of experimental and theoretical investigations including vibrations, charge distribution, energies of orbitals, and many more. Optimization results are then verified using experimental methods. In our study, we used optimization procedures to determine structures of PPL9, harmonic frequencies, vibrational intensities, ^1^H NMR spectra, probable site of imine protonation by CSA and HOMO and LUMO orbital energies.

## 2. Experimental

### 2.1. Materials

All chemicals and reagents were obtained from Sigma-Aldrich and used as received.

#### 2.1.1. Synthesis of 4-[(Di-p-tolyl-amino)-benzylidene]-(5-pyridin-4-yl-[1,3,4]thiadiazol-2-yl)-imine (PPL9) 

2.24 mmole (0.399 g) of 2-amino-5-(4-pyridyl)-1,3,4-thiadiazole and 10 mole percent (0.0386 g) of *p*-toluenesulfonic acid was added to 1.74 mmol (0.525 g) of 4-(di-p-toliloamino)benzaldehyde. The reaction was conducted at the temperature of (423–433) K for 65 h (see Figure S1). Then, the reaction mixture was dissolved in 10 mL dimethylacetamide. The mixture obtained was poured into 200 cm^3^ of water. The precipitant obtained was filtered off and air-dried. The product obtained was then dissolved in (10–12) mL of acetone using hotplate, and afterwards pipetted into a beaker containing 50 mL of cold (277) K hexane. Subsequently, the product was left to crystallize. The process was repeated twice [57]. Crystallized form of 4-[(di-p-tolyl-amino)-benzylidene]-(5-pyridin-4-yl-[1,3,4]thiadiazol-2-yl)-imine was obtained after 24 h, the structure of which was confirmed via single crystal X-ray diffraction. 

Yield: 34%. ^1^H NMR (400 MHz, CDCl_3_), δ [ppm]: 8.83 (1H, s, -N=CH-), 8.72 (2H, d, =CH-N-CH= pyridine); 7.82–7.75 (6H, m, arom. -Ph- and >C-(CH)_2_ = pyridine); 7.16–6.93 (10 H, arom. -Ph); 2.35 (6H, s, aliphatic). FT-IR [cm^−1^]: 3027vw, 2919vw, 2856vw, 1604w, 1573m, 1542m, 1501vs, 1433m, 1408m, 1398m, 1363w, 1320m, 1292m, 1274m, 1238m, 1215w, 1190w, 1159vs, 1110m, 1065w, 1040w, 1017w, 1001w, 989w, 967w, 919w, 886w, 833m, 814s, 784w, 765m, 728w, 713w, 695m, 653m, 638w, 610m, 595w, 581m, 565m, 554w, 522s, 511s, 497m, 490m, 454m, 421m, 416m, 410m, 393w, 385w, 374vw, 359w, 345m, 329w, 320m, 298w, 288vw, 277w, 264w, 252vw, 234w, 229vw, 207m, 175vw, 171vw, 159m, 144m, 130w, 121m, 110m, 103w, 99w, 95w, 91w, 86w, 78m, 72m, 69m, 67m, 64m, 60m. FT-Raman [cm^−1^]: 3064vw, 3037vw, 2922vw, 2789vw, 2556vw, 2520vw, 1615, 1605, 1581s, 1575s, 1552s, 1544s, 1502m, 1432m, 1409m, 1397vs, 1366m, 1327m, 1296w, 1275w, 1249m, 1240m, 1214w, 1191m, 1181m, 1166m, 1113m, 1085w, 1016w, 990w, 943vw, 916vw, 888vw, 863vw, 827w, 803m, 792w, 767w, 729w, 697w, 665w, 654w, 616w, 566w, 513w, 490w, 412m, 362w, 346w, 319w, 264w, 226w, 208vw, 112m (s—strong, m—medium, w—weak, w—weak, v—very); m. p. 194 °C, T_5%_ at 321 °C.

#### 2.1.2. Protonation of Imine PPL9

Protonation of PPL9 with CSA (dopant) was investigated in chloroform solution at room temperature. Dopant was added to the imine in chloroform solution in the 1:1 molar ratio. It was expected that protonation will occur on the most alkaline nitrogen of the pyridine moiety as N atoms of thiadiazole are more acidic. The estimated partial atomic charges on the pyridine N atom and the N atoms of thiadiazole were compared using natural bond orbital (NBO) analysis implemented in Gaussian 16 (see below) confirmed that the partial atomic charges on the pyridine N and thiadiazole nitrogen atoms are −0.405e and −0.292e and −0.267e, respectively. 

### 2.2. Methods

#### 2.2.1. X-ray Crystallography

For the single crystal of PPL9, X-ray data were collected by means of a KM4CCD diffractometer (Rigaku Oxford Diffraction, CrysAlisPro Software System, Rigaku Oxford Diffraction, Wroclaw, Poland). Empirical absorption corrections were carried out with the SCALE3 ABSPACK algorithm (implemented in CrysAlisPro). The crystal structure of PPL9 was determined using the SHELXT, SHELXL, and Olex2 programs [58,59]. All non-hydrogen atoms were refined anisotropically. Hydrogen atoms in methyl groups were treated as riding and rotating with U_eq_(H) = 1.5U_eq_(C), and the remaining H atoms were refined independently. The least-square refinement of the structure was carried out using F^2^. CCDC 2062220 contains the supplementary crystallographic data for this paper.

#### 2.2.2. Hirshfeld Surface Analysis

Hirshfeld surface maps and 2D fingerprint plots were prepared using CrystalExplorer17 [60] enabling quantitative estimation of percentage contributions for various intermolecular contacts in the reported crystals of PPL9 (trans-PPL9).

#### 2.2.3. NMR Spectroscopy

Sample was characterized with ^1^H NMR, using deuterated CDCl_3_ as a solvent with a Jeol ECZ-400 S spectrometer (^1^H-400 MHz) (Joel, Tokyo, Japan), the delay time of which was 5 s. 

#### 2.2.4. Vibrational Spectroscopy

Infrared (ATR FT-IR) spectra were measured on a Bruker Vertex 70v Fourier transform infrared spectrometer (Billerica, MA, USA) equipped with a diamond ATR cell (wavenumber range: 400–4000 cm^−1^ and 600–40 cm^−1^; resolution: 4 and 2 cm^−1^). 

Raman spectra were collected on a Bruker MultiRam FT spectrometer (Billerica, MA, USA) equipped with a liquid nitrogen cooled germanium detector and Nd:YAG laser emitting radiation at a wavelength of 1064 nm (laser power equal 450 mW, resolution 2 cm^−1^). 

#### 2.2.5. Thermal Analysis

Thermogravimetric analysis (TG-DTA) and differential scanning calorimetry (DSC) were performed on LABSYS EVO 1150 °C TG-DTA/DSC instrument (TAL, Fredericton, N.B., Canada) using alumina pans under nitrogen atmosphere with heating rate of 5 °C/min.

#### 2.2.6. UV Spectroscopy

Absorption spectra of imine in neutral and protonated form in chloroform were recorded using spectrometer UV–vis model A360 (AOE Instruments, Shanghai, China).

#### 2.2.7. Electrochemical Analysis

MetrohmAutolab PGSTAT M204 potentiostat (Barendrecht, the Nederland) was used to carry the electrochemical measurements. The electrochemical cell consisted of a glassy carbon electrode (with diameter of 2 mm), a platinum rod and Ag/AgCl electrodes. The ferrocene (Fc) served as the potential reference indicator and the internal standard. The voltammograms were recorded in a standard single-compartment electrochemical cell in acetonitrile (purity ≥ 99.9%, Honeywell, Charlotte, NC, USA), under argon. In all experiments as a supporting electrolyte 0.2 M Bu_4_NPF_6_ solution (purity 99%, Alfa Aesar, Haverhill, MA, USA) was used. The argon was bubbled through the system for about 15 min prior to the measurement to achieve deaeration of the solution The electrochemical study was undertaken at ambient temperature and atmospheric pressure for PPL9 with concentration 1.0 × 10^−6^ mol dm^−3^. The electrochemical measurements were recorded at the scan of 100 mVs^−1^ and voltage step 0.00244 V.

#### 2.2.8. Thermal Imaging

The thermal images were registered for the samples connected to bias voltage in the range of 0 to 10 V using thermographic camera (VIGOcam v50, VIGO System S.A, Ożarów Mazowiecki, Poland). To control the exact potential value and the electric response a multichannel potentiostat-galvanostat (PGStatAutolab M101, Metrohm, Barendrecht, the Nederland) was employed and controlled by computer software Nova 2.0. The experimental design include application of potential in three-minute time window and 0.5 V step in whole range. The samples preparation included layer formation by spin-coating technique (5000 rpm for 20 s) using dichloroethane solution (15 mg/mL) The detailed description of the experiment was reported in our previous work [61].

#### 2.2.9. Theoretical Calculations

The molecular geometry extracted from the crystal structure was used as a starting point in the optimization procedures. Full geometry optimizations were performed for monomer PPL9 (*trans*-PPL9). The harmonic frequencies, infrared intensities and Raman scattering activities were calculated for C1 symmetry. In the calculations, we used the Becke three-parameter hybrid functional, B3LYP [62,63] combined with the 6-31G(d,p) basis set. The computations were performed with the Gaussian 09 set of programs [64]. The theoretical Raman intensities (which simulate the measured Raman spectrum) were calculated according to the formulas reported by Michalska and Wysokinski [65]. The rigorous normal coordinate analyses for PPL9 were carried out and the potential energy distributions (PEDs) were calculated using the FCART07 program [65,66]. Normal vibrations and corresponding IR and Raman bands were characterized on the basis of PED results and additionally verified by atom displacement animation done by Chemcraft program (https://www.chemcraftprog.com, accessed on 3 February 2021) and the Gauss-View program [67].

Theoretical calculations (HOMO-LUMO) were performed using Gaussian 16 [61,67] software package. The structures of the investigated imine were optimized using density functional theory (DFT) and B3LYB exchange-correlation functional [62,63,68] with the 6-31G(d,p) basis set, to a stationary point on the Born–Oppenheimer potential energy surface proved by the absence of imaginary frequencies. The frontier HOMO and LUMO energies were calculated on fully optimized structures by employing both the DFT/B3LYP 6-31G(d,p) and TD-DFT/B3LYP 6-31G(d,p) [69] approaches.

## 3. Results and Discussion

### 3.1. Synthesis of PPL9

The PPL9 imine was synthesized in an one-step condensation reaction in molten-state between of 2-amino-5-(4-pyridyl)-1,3,4-thiadiazole and 4-(di-p-toliloamino)benzaldehyde (see Figure S1 in Supplementary Materials). Details about the synthesis are presented in the experimental part. The purification progress was monitored by proton nuclear magnetic resonance (^1^H NMR) and thin layer chromatography (TLC). The PPL9 as crystal was obtained in low yield (~34%) and was characterized using X-ray diffraction, ^1^H NMR, FT-IR, FT-Raman spectroscopy and cyclic voltammetry (CV). The molecular structure of trans-PPL9 was confirmed by X-ray diffraction, vibration spectra (FT-IR, FT-Raman) and ^1^H NMR (see below). Therefore, the final conclusion is that the compound is obtain in the unique trans form as confirm by SCXRD and NMR studies.

### 3.2. Crystal and Molecular Structure of Trans-PPL9

**Figure 1:**
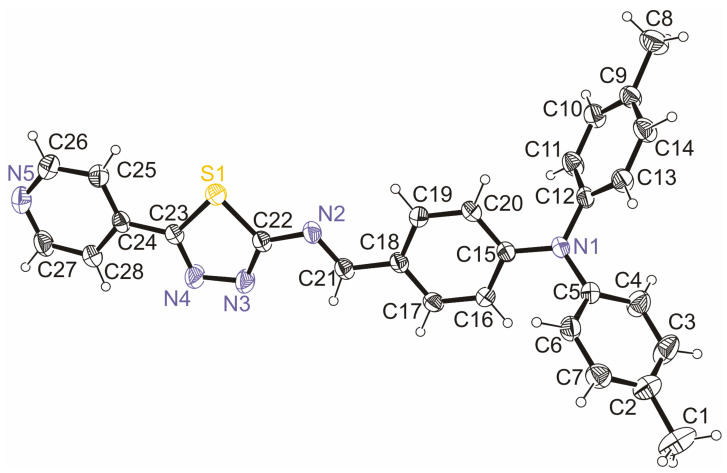
Asymmetric unit for *trans*-PPL9 with the numbering scheme, the atomic displacement parameters for the non-hydrogen atoms were drawn at the 35% probability level.

**Figure 2:**
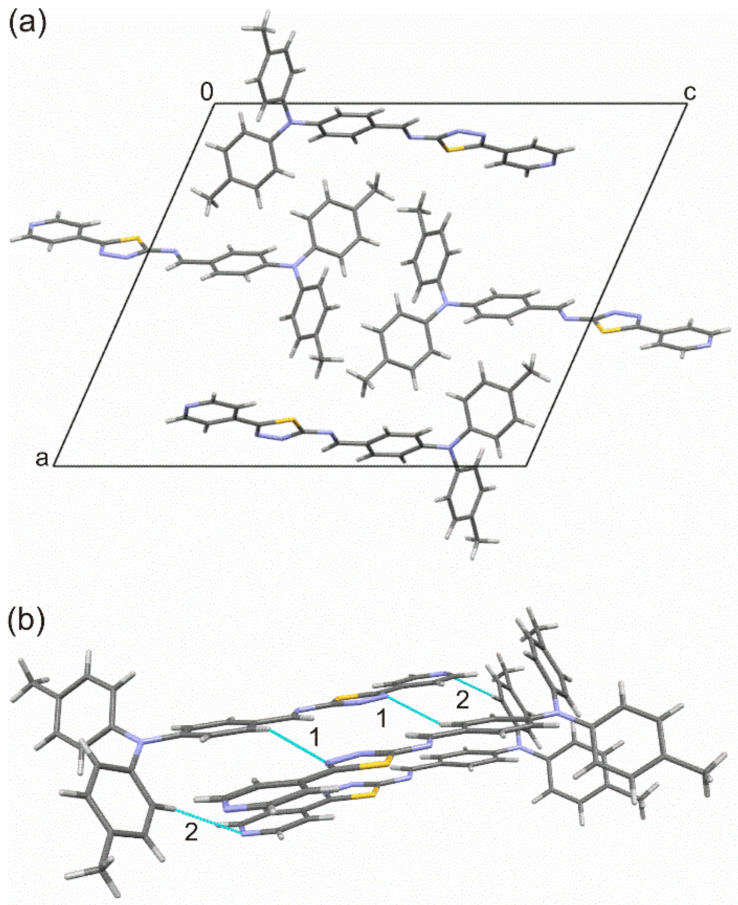
(**a**) Unit cell of PPL9, (**b**) intermolecular interactions for PPL9, where (1) is C17−H17⋯N4 and (2) is C6−H6⋯N5.

**Figure 3:**
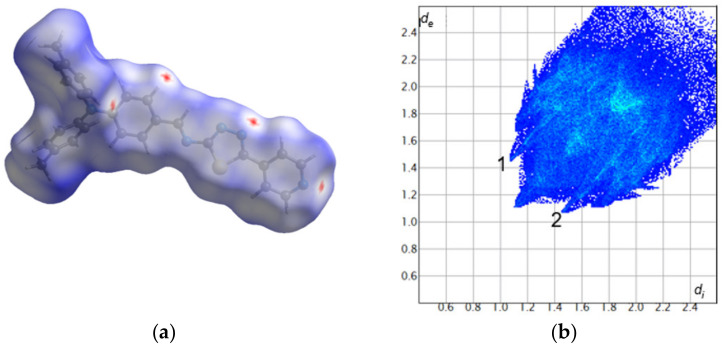
Hirshfeld surface (**a**) and fingerprint plot (**b**) for PPL9. The red, white, and blue colors on the Hirshfeld surface indicate contacts shorter, equal, and longer than the sum of the van der Waals radii, respectively. *d_e_* and *d_i_*—distances from a point on the surface to the nearest atom outside and inside the surface, respectively.

The molecular structure and atom-numbering scheme for PPL9 are shown in Figure 1. What results from the X-ray structure analysis is that the molecule of PPL9 exists as the trans isomer and is planar from N1 atom towards N5, and the rmsd (root-mean-square deviation) of atoms from this plane is 0.05 Å. The crystal unit cell contains four molecules (Figure 2a), but only one molecule is symmetrically independent. Intermolecular interactions in the crystal shorter than the sum of the van der Waals radii are only weak C−H⋯N hydrogen bonds (Figure 2b) shown by the Hirshfeld surface (Figure 3). The geometry of the intermolecular interactions is given in Table 1. The hydrogen bonds form zigzag ribbons along the *b* axis. The flat fragments N1→N5 of neighboring molecules form stacking. The geometry of π⋯π interactions is presented in Table 1. In Table 2, selected crystallographic and experimental data concerning PPL9 are presented. Additionally, bond lengths and bond angles determined for PPL9 by X-ray diffraction and the corresponding theoretical parameters, calculated for PPL9 by B3LYP/6-31G(d,p) method are shown in Table S1 of the Supplementary Materials.

The Hirshfeld surface analysis was used to further study the intermolecular interactions in the PPL9 crystal structure. The Hirshfeld surface mapped with d_norm_ is shown as transparent to allow visualization of the molecular moiety around which it was calculated (Figure 3). The deep red spots indicate the intermolecular hydrogen bonding interactions. The hydrogen bonds are also evidenced by two spikes (1 and 2) in the fingerprint plot. The relative contributions to the overall Hirshfeld surface come from the following close contacts: H⋯H (51.0%), C⋯H/H⋯C (21.9%), N⋯H/H⋯N (14.6%), S⋯H/H⋯S (5.9%), C⋯C (3.1%), N⋯C/C⋯N (2.5%), S⋯N/N⋯S (0.7%), and N⋯N (0.3%). In Figure S2 of the Supplementary Materials we presented the front and back views of the three-dimensional Hirshfeld surface mapped with d_norm_ along with the shape index and curvedness surfaces of PPL9.

**Figure 4:**
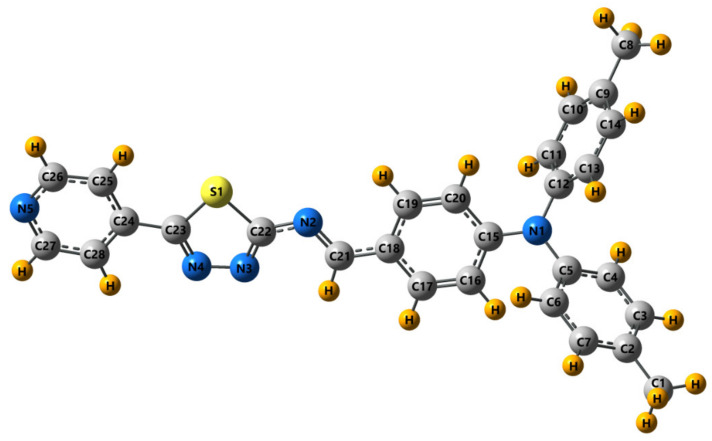
DFT-optimized molecular geometry of PPL9 obtained by B3LYP/6-31G(d,p).

To evaluate the accuracy of the predicted geometrical parameters, we have calculated the overall mean percent deviations (Ds), as suggested in [70]. Deviations values between the experimental and the theoretical bond lengths in PPL9 (omitting the C-H bond lengths) is equal to 1.35%. The Ds values for the calculated bond angles (neglecting those involving the H atoms) in these molecules are 0.43%. Optimized molecular geometry of PPL9 with atom numbering scheme is presented in Figure 4. It should be stressed, that our results put into light possibility of monocrystal formation for a molecule regardless the presence of bulky triphenylamine (TPA) [52]. The TPA does not suppress the crystallinity but also is involved via hydrogen bonding with nitrogen N4 of pyridine and N5 of thiadiazole ring.

### 3.3. Spectroscopy

As stated above, the crystal structure from X-ray diffraction clearly confirm the trans isomer. The ^1^H NMR spectra showed that only one isomer was obtained and it was assumed as the trans form, known from the literature as the naturally preferable one [71].

Representative experimental infrared and Raman spectra of PPL9 appear in Figures S3–S6. The observed positions of vibrational bands and their assignments are summarized in Table 4 for PPL9. Details in the Supplementary Materials). In the PPL9 infrared spectrum (Figure S3) characteristic 1683 cm^−1^ band, which is responsible for -CHO stretching and which is visible in the measured aldehyde IR spectrum, has not been observed, and neither have -NH_2_ stretching bands in the amine. Nevertheless, new bands (HC=N-) appeared, which point to the formation of a new compound—an imine. As far as experimental spectrum is concerned, this occurs at 1573 cm^−1^ (as medium band), whereas in the Raman spectrum—at 1581 and 1575 cm^−1^ (as medium band). The most significant contribution of this vibration takes place (in theoretical study), when the band length equals 1631 cm^−1^. DFT calculation point to these vibrations also taking place at 1664 and 1588 cm^−1^, however their contribution is much lower. In the experiment, this corresponds to 1604 and 1542 cm^−1^ (as far as IR is concerned) and 1615, 1606, 1552, and 1544 cm^−1^ (in case of Raman spectra). Detailed vibrational band assignment, with regard to all the remaining bands, is presented in Table 3. Good agreement between theoretical and experimental results was obtained.

New bands (HC=N-) is observed also in ^1^H NMR spectra (see Figure S7). In proton NMR spectrum of the investigated PPL9, the imine proton signal was observed at 8.83 ppm.

### 3.4. Theoretical and Experimental Cyclic Voltammetry Study of PPL9 and Their Protonated Form

**Figure 5:**
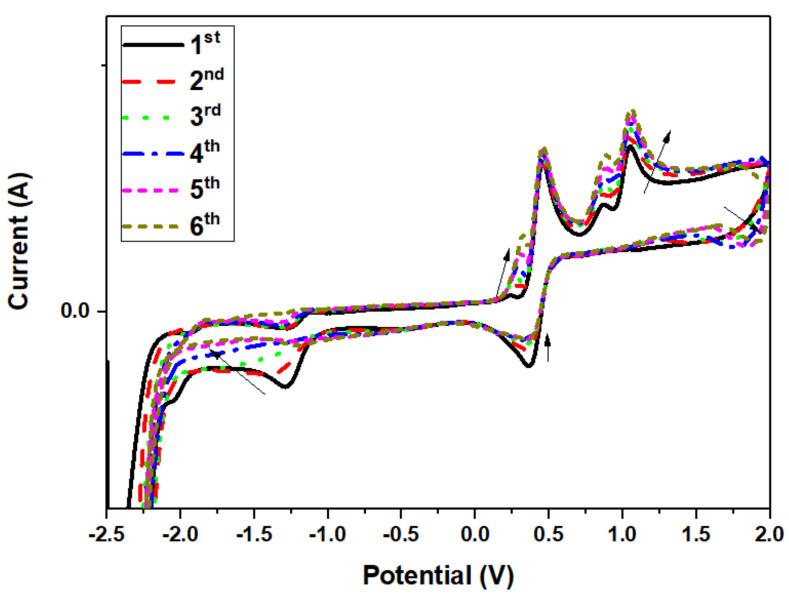
Cyclic voltammograms of PPL9: six consecutive scans at scan rate 100 mV·s^−1^.

Electrochemical studies, namely concerning oxidation and reduction potentials, were used to calculate the HOMO-LUMO ionization levels of PPL9, using ferrocene’s potential (−5.1 eV) as reference. HOMO of PPL9 was found at −5.41 eV, while that of LUMO—at −2.52 eV. Figure 5 presents cyclic voltammograms of PPL9 in acetonitrile with Bu_4_NPF_6_ as supporting electrolyte at constant scan rate.

For PPL9, several oxidation and reduction processes can be observed in cyclic voltammogram. Apart from an onset of the oxidation process at 0.96 V, four maxima were distinguished at −1.25 V, 0.26 V, 0.88 V, and 1.05 V. On the other hand, the reduction process exhibited reduction offset at −2.05 V, and two noticeable maxima—at −1.35 V and 0.35 V. Taking the molecular structure of imine the processes observed at −1.25 V and 1.35 V corresponding to oxidation and reduction were assigned to pyridine unit. Lucio et al. [72] observed in their work that pyridine molecule exhibited a single-electron process of irreversible reduction at −1 V. Similar shape of the curve was observed in the case of PPL9 at much lower potentials, −1.35 V. Moreover, regarding another molecule segment, namely TPA, two oxidation maxima at 0.88 and 1.05 V could be assigned, based on the research by Chiu et al. [73], who reported two one-electron oxidation steps resulting in formation of monopositive cation-radical and dipositively charged TPA. Evaluating the oxidation-reduction stability based on the changes observed on consecutive scans at the same scan rate, it can be noticed that the oxidation-reduction processes are fully reversible formation continuous loops for succeeding scans. Nevertheless, some changes in the shape of peaks were noticeable; the intermediate reduction processes showed tendency to reduce their signal at −1.35 V and 0.35 V with subsequent formation of new signal at approx. 1.76 V. In the oxidation scan, increase of the following signals was observed: 0.26 V, 0.88 V, and 1.05 V. It is most likely that the changes in the concentration of specific species formed during oxidation-reduction processes could be affected by absorption of oxidized form on the electrode surface, enabling flow of molecules in the solution.

**Figure 6:**
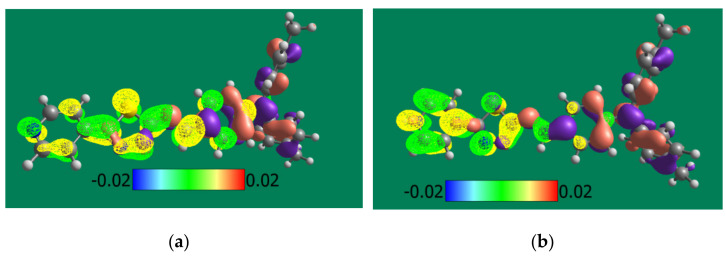
Modeled HOMO (brown and purple) and LUMO (yellow and green) orbitals for *trans*-PPL9 (**a**) and H^+^ doped *trans*-PPL9 (**b**) molecule. Brown and yellow lobes correspond to positive-, whereas purple and green lobes—to negative isosurface values.

Model of HOMO and LUMO orbitals for both optimized isomer molecules in undoped and doped states are shown in Figure 6.

The experimental values of HOMO and LUMO energies of PPL9 were compared with the theoretical ones, calculated by B3LYP/6-31G(d,p) method. As visible, the electron density of HOMO level of imine is located mainly on triphenylamine moieties (brown and purple in Figure 6) and extended to imine bond. The LUMO (yellow and green) is located only on pirydinyl thiophene moieties. For *trans*-PPL9 the LUMO-HOMO energy gap of PPL9 from DFT/B3LYP/6-31G(d,p) was calculated to be 2.71 eV, whereas for *cis* isomer it equalled 3.54 eV. The TD-DFT/B3LYP/6-31G(d,p) method gave 3.00 eV and 3.12 eV, respectively (see Table 4).

Finally, it can be concluded that for trans-PPL9 the experimental *Eg* 2.89 eV from the CV experiment is quite consistent with the theoretically calculated results both by the time dependent method and the time independent DFT method, whereby the former performs slightly better. For investigated imine PPL9, a good correlation was found between the LUMO levels determined on the basis of CV and DFT/B3LYP/6-31G (d, p) (−2.52 eV and −2.46 eV, respectively).

As can be seen in Table 4, the H^+^ doping of PPL9 forms theoretically a significant impact on the energies of HOMO and LUMO orbitals, in particular the reduction of the energy of the LUMO level (>3 eV), which in turn should lead to an increase in the electron-acceptor properties of the doped molecule. Taking the DFT results into account (Figure 6), the LUMO of the doped imine is located predominantly on pyridine ring while the HOMO is placed on the triphenylamine fragment. Therefore, it seems that the analysis of the frontier orbitals can be helpful in understanding changes in the photo-optical activity of the doped PPL9 imine as imines are an interesting material for active layers for organic photovoltaics and their H-doping is a way to manipulate the energy levels of HOMO LUMO frontier orbitals responsible for the efficiency of solar cells built from it.

### 3.5. Proton NMR Study of PPL9:CSA(+) and PPL9:CSA(−)

**Figure 7:**
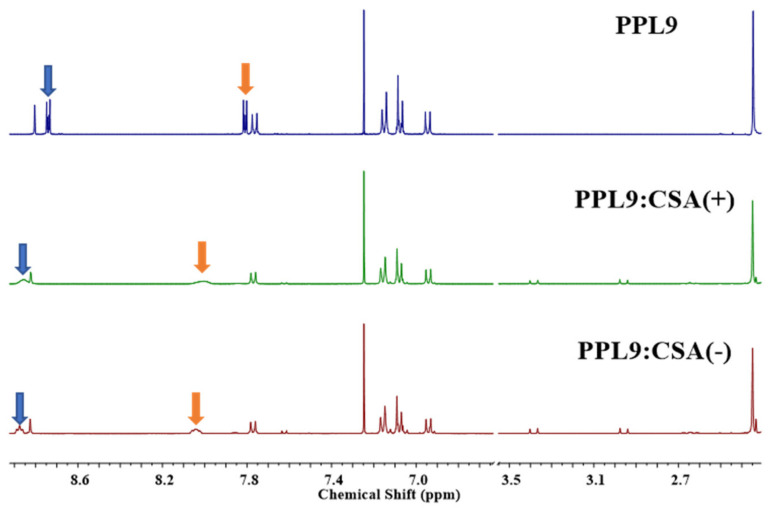
^1^H NMR of PPL9 and PPL9 with CSA(−) and CSA(+) in 1:1 molar ratio in CDCl_3._

In order to establish which of the nitrogen atoms participates in the formation of complexes in the solution (CDCl_3_), we performed the ^1^H NMR study where to PPL9 equimolar portion of 10-camphorosulfonic acid in one of the racemic forms were added. In case of PPL9 protonated with CSA(+) and CSA(−), the proton spectra are found very similar and no differences between the used racemic forms was observed, showing only signals coming from the both compounds: 0.8, 1.1, 1.3, 1.8, 2.0, 2.3, 2.8, 2.9, 3.4 ppm, corresponding to CSA molecule and 7.0–7.4, 7.5–7.6, 8.31 ppm assigned to PPL9 (Figure 7), as expected. The only noticeable difference in the PPL9 spectra as a consequence of the protonation was the chemical shift of the pyridine protons in mixtures with CSA towards higher values. It was evidenced that both protons from position 2 and 6 (doublet at 8.72 ppm) and also 3 and 5 (doublet at 7.80 ppm) shifted of about 0.2 ppm, and the signal got unified and very broad, for both pairs of protons.

Theoretical ^1^H NMR spectra of the *trans*-PPL9 undoped and doped isomer are presented in Figure S8. Protonation of pure pyridine moves the proton NMR chemical shift to the down field (Wiley SpectraBase; http://spectrabase.com/ (accessed on 21 November 2020)).

Analysis of the obtained both experimental and theoretical ^1^H NMR spectra shows similar effects for the pyridine ring protons in PPL9. Interestingly, in the case of the estimated spectra, the greatest change in the chemical shift is observed for protons adjacent to the thiadiazole ring (see 10-H, 54-H, Figure S8), which suggests strong ‘trough space’ interactions of the -N=N- group with these protons. The effect of H doping on the protons of the triphenylamine group is relatively small and more complex. Concluding, due to hindrance of thiadiazole ring and bigger accessibility of pyridine atom and greater acidic nature the protonation occurred on pyridine moiety affecting only signals coming from pyridine hydrogen in proton nuclear resonance spectroscopy.

### 3.6. Thermal Imaging of PPL9 and PPL9:CSA

**Figure 8:**
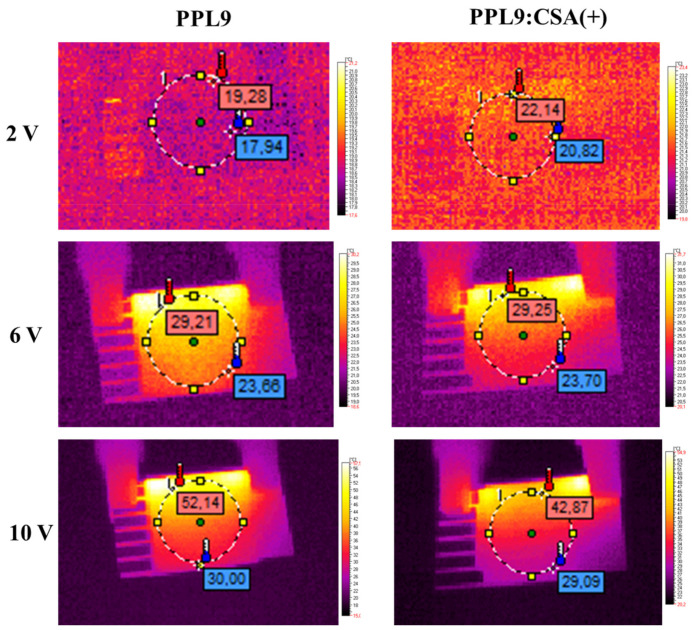
IR images obtained for constructed devices at 2 V, 6 V, and 10 V.

To investigate the thermal behavior of an organic layer composed of PPL9 or PPL9:CSA(+) were prepared by spin coating method and a simple devices with following architecture ITO/PPL9/Ag/ITO or PPL9:CSA(+)/Ag/ITO was made. Taking into consideration the fact that not spectacular differences in UV–vis study was found along with change CSA(+) to CSA(−) in this part of our work we investigated only CSA(+) as an additive in equimolar ratio to PPL9. Generally, the comparison of thermal images containing different organic layer composition put into evidence very similar thermal response to passing current (see Figure 8). The heating was initiated at places of contact between the metallic clamps and conducting glass. When the current increased, it started to be more noticeable that the heating zone has been concentrated at upper part of the constructed device. It can be caused by heating of the part where the current flow was the highest. 

**Figure 9:**
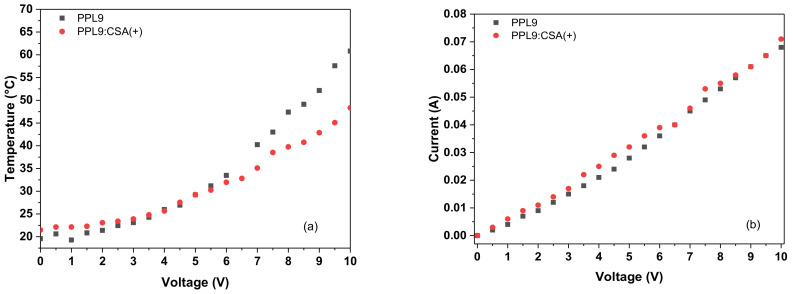
Correlation of temperature (**a**) and current (**b**) versus applied potential for constructed devices containing PPL9 and its protonated form with CSA(+).

In both cases, PPL9 and PPL9 with CSA(+) the thermal response to passing current had logarithmic shape overlapping up to 6 V (Figure 9a). Above this value the temperature for PPL9 had slightly steeper than in the mixture with CSA(+), giving final value of 60.8 °C and 48.3 °C, respectively. Normalized resistance to 1 cm^2^ layer area was very similar around 139 Ω. The current passage in both cases was linear in all range of voltage and was almost overlapped for both samples as it can be seen in Figure 9b. The addition of acidic dopant into active layer lowered the maximal observed temperature of tested devices of about 12 °C, which gave a substantial advantage in terms of diminishing the overheating effect caused by current flow. It is widely known that elevated temperature has a negative influence on performance of organic devices [1,4,63].

### 3.7. Thermal Stability PPL9

For investigated imine by TGA two main reaction stages was found. The initial decomposition based on 5% weight loss occurred, which is usually considered the criterion for assessing the thermal stability, lied at 321 °C (see Figure S9). The TGA analysis suggested good thermal stability in nitrogen atmosphere. After the second step of decomposition at 800 °C, the char yield percentage in the imine PPL9 was 40%. 

### 3.8. Protonation of PPL9 by CSA: UV–Vis Study

Finally, we investigated influences of CSA in both racemic form in various ratio on UV–vis absorption properties of PPL9. Protonation of PPL9 with CSA(+) and CSA(−) was carried out at room temperature using chloroform as a solvent. Dopant was added to the chloroform solution of imine studied in the 1:1, 1:2, 1:3 ratio with respect to nitrogen atoms.

**Figure 10:**
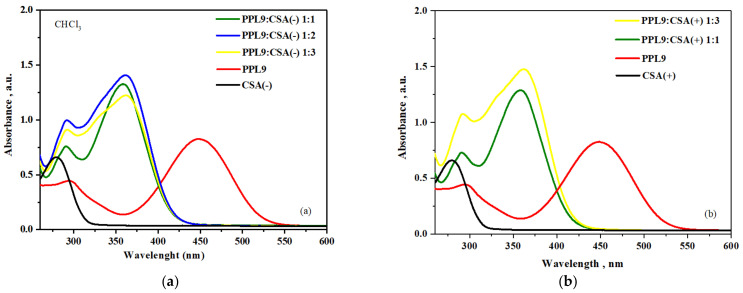
UV–vis study of PPL9 and their mixtures with CSA (−) (**a**) and CSA (+) (**b**).

The PPL9 and PPL9:CSA compositions were investigated in chloroform by UV–vis spectroscopy in various concentration of CSA(+) and CSA(−) monitoring the changes in the π–π * and n–π * transitions. The solution of investigated PPL9 imine showed two absorption bands with maxima peaks at 297 nm and 448 nm (see Figure 10). In chloroform solution, all investigated PPL9 compositions with CSA exhibited blue shift of the absorption band maximum (responsible for the π–π * transition in the imine group) in comparison with the pure PPL9.

Maximum of absorption band of all PPL9:CSA compositions was about 88 nm blue shifted compared with pure PPL9 (from 448 nm to about 360 nm). No changes were observed along with increase concentration of CSA and used racemic form of CSA. Moreover, in UV–vis spectra of the PPL9:CSA compositions the hyperchromic effect was observed compared with PPL9. An advantage of such blue shit for CSA-doped give possibility to use this material as admixture to other active component like PTB7, having main absorption maxima at 621 and 680 nm, being complementary in absorption range replacing the currently used fullerene derivatives.

As our calculations showed, after protonation by proton on pyridine atom in imine PPL9 both HOMO-LUMO levels changed a lot and as a consequence E_g_ value for protonated PPL9 imine decreased (see Table 4 and Figure 6). This part of our work needs more investigations.

## 4. Conclusions

In conclusion, the new unsymmetrical imine was successfully obtained by the condensation reaction of 4- (di-p-tolylamino) benzaldehyde with 2-amino-5-(4-pyridinyl)-1,3,4-thiadiazole. The single crystal XRD studies of *trans*-PPL9 have shown that the imine crystallizes in the monoclinic system (space group P2_1_/n). According to the single-crystal X-ray diffraction, it was established that the crystal structure of PPL9 was stabilized by intermolecular hydrogen bonds C−H⋯N, as shown by the Hirshfeld surface analysis, despite the presence of bulky TPA unit. The FT-Raman and FT-IR spectra of PPL9 were reported, for the first time. Full geometry optimization of PPL9 was performed by the B3LYP/6-31G(d,p) method. The results showed good agreement with the experiment. Theoretical spectra (^1^H NMR and HOMO-LUMO energies) are in quite good correlation with the experimental data.

Moreover, complexes of imine with CSA in both racemic forms were obtained and investigated in depth by UV–vis in chloroform solution taking into consideration the weight ratio of both components used. In all investigated cases, independence of racemic forms of CSA using hypsochromic effect was found when compared with undoped PPL9 in chloroform solution. As the only NMR signals affected by the protonation are those of pyridine nitrogen atom. The protonation of PPL9 is then confirmed to occur only on pyridine and not on thiadiazole as expected from the greater acidic character of thiadiazole N atoms. Dysfunctionalities of the active layer based on imine and imine:CSA was analyzed by IR thermographic camera. The thermal imaging for simple devices built of one- or two-component layers showed a wider area where the temperature was higher. Use of CSA-doped imine in organic devices could give additional benefits, such as lowering the heating process caused by current flow, and complementary absorption spectra to most commonly used PTB7. Due to a great interest in this family of compounds, these results can be used in further study of new imines using in optoelectronics.

## Figures and Tables

**Table 1:** Geometry for the intermolecular interactions in PPL9.

Contact	D−H [Å]	H⋯A [Å]	D⋯A [Å]	<DHA [°]
C6−H6⋯N5^i^	0.92(2)	2.68(2)	3.498(3)	148(2)
C17−H17⋯N4^ii^	0.96(2)	2.64(2)	3.509(3)	152(2)
**Fragments**	**Cg1** **⋯** **Cg2 [Å]**	**D1 [Å]**	**D2 [Å]**	
C22→S1, C22^i^→S1^i^	3.701	3.538	1.086	
N1→N5, N1^i^→N5^i^	4.090	3.614	1.915	

Symmetry codes: (i) 1-x, 2-y, -z; (ii) 1-x, 3-y, -z; Cg1, Cg2—centroids of fragments, D1—interplanar distance, D2—centroid slippage.

**Table 2:** Selected crystallographic and experimental data for PPL9.

Chemical Formula	C_28_H_23_N_5_S
Formula weight	461.57
Crystal dimensions/mm	0.44 × 0.16 × 0.05
Temperature/K	298
Crystal system	Monoclinic
Space group	P2_1_/n
a/Å	18.9567(7)
b/Å	6.18597(17)
c/Å	22.5897(7)
β/°	114.009(4)
V/Å^3^	2419.81(15)
Z	4
D_x_/Mg m^−3^	1.267
μ/mm^−1^	0.160
Reflections collected	17048
Reflections independent	4762
Reflections ind. observed	3343
R_int_	0.032
R, wR (F^2^ > 2σ(F^2^)), S	0.054, 0.113, 1.03
Δρ_max_, Δρ_min_/eÅ^−^^3^	0.29, −0.36

**Table 3:** Comparison of the experimental infrared and Raman bands of PPL9 with the theoretical harmonic wavenumbers (ν^a^, cm^−1^) calculated for PPL9 by the B3LYP method with 6-31G (d,p) basis set.

FT-IR	FT-R	ν^a^	Assignments
n.o.	n.o.	3233	ν(CH)_pyridine_
n.o.	n.o.	3226	ν(CH)_s_^phe^
n.o.	n.o.	3225	ν(CH)_s_^phe^
n.o.	n.o.	3212	ν(CH)_as_^phe^
n.o.	n.o.	3210	ν(CH)_s_^arom^
n.o.	n.o.	3209	ν(CH)_s_^arom^
n.o.	n.o.	3208	ν(CH)_as_^arom^
n.o.	n.o.	3207	ν(CH)_as_^arom^
n.o.	n.o.	3199	ν(CH)_s_^pyridine^
n.o.	n.o.	3182	ν(CH)_as_^phe^
n.o.	n.o.	3180	ν(CH)_as_^arom^
n.o.	n.o.	3179	ν(CH)_as_^arom^
n.o.	n.o.	3179	ν(CH)_as_^arom^
3027vw	3064vw	3178	ν(CH)_as_^arom^
n.o.	n.o.	3166	ν(CH)_as_^pyridine^
n.o.	n.o.	3127	ν(CH_3_)_as_
2919vw	2922vw	3126	ν(CH_3_)_as_
n.o.	n.o.	3098	ν(CH_3_)
2856vw	n.o.	3038	ν(CH_3_)_s_
1604vw	1615vw 1606vw	1664	ν(CC)^arom^, ν(C=N)
1573m	1581m 1575m	1631	ν(C=N), ν(CC)^arom^
1542m	1552m 1544m	1588	ν(C=N), ν(CC)^arom^
1501s	1502m	1540	δ(CCH), ν(CN)^pyridine^
1433m	n.o.	1488	ν(CN), ν(CC)
n.o.	1432m	1478	ν(CN), ν(CC)
n.o.	1409vw	1459	δ(CCH), ν(CC)
1408m 1398m	1397vs	1455	δ(NCH), ν(CC), δ(CCH), ν(CN)^th^
		1442	ν(CN)^th^, ν(C=N), δ(CCH)
1363w	1366m	1404	ν(CN)^th^, δ(NCH), ν(C=N)
1320m	1327m	1360	ν(CN)^arom^, δ(CCH)
n.o.	1296w	1326	ν(CN)^arom^, ν(CC)
1292m	1249m	1297	ν(CN), ν(CC)
1274m	1240m	1277	ν(CC), δ(NCH)
n.o.	1214w	1250	δ(NCH), δ(CCH), ν(CN)
1215w	1181m	1207	δ(NNC), δ(CCH), ν(CN)
1190w	n.o.	1185	δ(CCH), ν(CN)
1159vs	1166m	1171	ν(NN), δ(NNC)
1110m	1113m	1144	δ(CCH)
833m	827w	839	γ(H(CCC)
814s	n.o.	838	γ(H(CCC)
n.o.	803m	821	ν(CN), δ(NNC), ν(CS)
n.o.	792w	804	ν(CS), ν(CC), δ(NNC)
784w	n.o.	796	ν(CC)^arom^, δ(CCC)
765m	767w	765	ν(CS), δ(NNC), ν(CC)
728w	729w	729	τ(CCCC), γ(C(CCC)
713w	665w	700	δ(CNC), ν(CS)
695m	n.o.	662	δ(CCC), ν(CS)
n.o.	654w	637	τ(SCNN), τ(CNNC), τ(SCNN)
653m	638br	624	δ(CSC), δ(SCN)
565m	n.o.	575	δ(CCC), ν(CC)
522s	n.o.	540	γ(C(CNC)^pyridine^, γ(C(CCC)^phe^
511s	525vw	524	γ(C(CNC), τ(CCNC)
497m	490w	495	γ(C(CCC), ν(CS) δ(CCC)
454m	421m	431	τ(CCCC)^phe^
374vw	362w	356	δ(CCC)
345m	346w	342	γ(C(CCC), τ(CCCN)
n.o.	319w	322	δ(NCC), τ(NCCC)
n.o.	264w	221	τ(CCCC), τ(NCCC)

Abbreviations: ν—stretching; δ—in-plane bending; γ—out-of-plane bending; τ—torsion; s—strong; m—medium; w—weak; w—weak; v—very; pyridine—pyridine ring; phe—phenyl ring; th—thiadiazole ring; arom—aromatic. n.o.—not observed; _as_—antisymmetric; _a_—symmetric; ^a^—calculated wavenumbers were non scaled.

**Table 4:** Comparison of theoretical and experimental values of *E_g_* and energies of HOMO LUMO levels for *trans*-PPL9 in undoped and doped state (1:1 *w*/*w*).

Frontier Orbital Energy at DFT/B3LYP/6-31G(d,p)			
	HOMO [eV]	LUMO [eV]	*Eg* [eV]
*trans*-PPL9	−5.17	−2.46	2.71
H^+^ doped *trans*-PPL9	−7.07	−5.95	1.12
**TD-DFT/B3LYP/6-31G(d,p)**			
*trans*-PPL9	−5.26	−2.26	3.00
H^+^ doped *trans*-PPL9	−5.75	−7.35	1.60
**Cyclic Voltammetry**			
PPL9	−5.41	−2.52	2.89

## Data Availability

The data presented in this study are available in Supplementary Materials and on request from the corresponding author.

## Supplementary Materials

The following are available online at https://www.mdpi.com/article/10.3390/ma14081952/s1, Figure S1: Photos of obtained crystals PPL9 and scheme of imine formation: aminocarbinol formation (left) and imine formation (right). Figure S2: View front (left) and back (right) of the three-dimensional Hirshfeld surface present views of d_norm_, shape index and curvedness surfaces of PPL9. Figure S3: Experimental FT-Raman spectra of crystalline PPL9 range 3600–400 cm^−1^. Figure S4: Experimental FT-IR spectra of crystalline PPL9 range 4000–400 cm^−1^ (red) and range 600–50 cm^−1^ (green). Figure S5: Theoretical scaled Raman spectra of PPL9 in the range 3500-50 cm^−1^. Figure S6: Theoretical scaled FT-IR spectra of PPL9 in the range in the range 3500–50 cm^−1^. Figure S7: Experimental ^1^H NMR spectra of crystalline PPL9. Figure S8: Simulated ^1^H NMR spectrum of trans-PPL9 according to GIAO method at DFT/B3LYP/6-311+G(2d,p) level. Numbering of trans-PPL9 (H^+^ doped) (a) ^1^H NMR spectrum of trans-PPL9 (b) ^1^H NMR spectrum of H^+^ doped trans-PPL9 (c). Figure S9: TGA of PPL9. Table S1: The bond lengths (Å) and bond angles (°), with s.u.s. in parentheses, determined for PPL9, by X-ray diffraction and the corresponding theoretical parameters, calculated for trans-PPL9 by B3LYP method 6-31+G (d,p) basis set. Table S2: A statement of the theoretical harmonic wavenumbers (ν^a^, ν^b^, cm^−1^), infrared intensities (A^IR^, km mol cm^−1^), Raman scattering activities (S^R^, A^4^ amu cm^−1^) and Raman intensities (IR) calculated for PPL9 by the B3LYP method with 6-31+G (d,p) basis set.
