# Supplementary material for: Crystal Structure Determination of 4-[(Di-p-tolyl-amino)-benzylidene]-(5-pyridin-4-yl-[1,3,4]thiadiazol-2-yl)-imine along with Selected Properties of Imine in Neutral and Protonated Form with Camforosulphonic Acid: Theoretical and Experimental Studies
Source: Materials (Basel). 2021 Apr 13;14(8):1952. doi: 10.3390/ma14081952 (PMC8070542; doi:10.3390/ma14081952)
Supplement: Supplementary file 1 [file materials-14-01952-s001.pdf]

materials

MDPI

Supplementary Materials

Crystal Structure Determination of 4-[(di-p-tolyl-amino)-benzylidene]-(5-pyridin-4-yl-[1,3,4]thiadiazol-2-yl)-imine along with Selected Properties of Imine in Neutral and Protonated Form with Camforosulphonic Acid: Theoretical and Experimental Studies

Agnieszka Dylong [1], Karolina Dysz [1], Krzysztof A. Bogdanowicz [1], Wojciech Przybył [1], Krzysztof A. Konieczny [2], Ilona Turowska-Tyrk [2], Andrzej Kaim [3], and Agnieszka Iwan [1,*]

[1] Military Institute of Engineer Technology, 136 Obornicka str, 50-961 Wroclaw, Poland; dylong@witi.wroc.pl (A.D.); dysz@witi.wroc.pl (K.D.); bogdanowicz@witi.wroc.pl (K.A.B.); przybyl@witi.wroc.pl (W.P.)
[2] Faculty of Chemistry, Wrocław University of Science and Technology, 27 Wybrzeże Wyspiańskiego, 50-370 Wroclaw, Poland; krzysztof.konieczny@pwr.edu.pl (K.A.K.); ilona.turowska-tyrk@pwr.edu.pl (I.T.-T.)
[3] Faculty of Chemistry, University of Warsaw, 1 Pasteura str., 02-093 Warsaw, Poland; akaim@chem.uw.edu.pl
* Correspondence: iwan@witi.wroc.pl

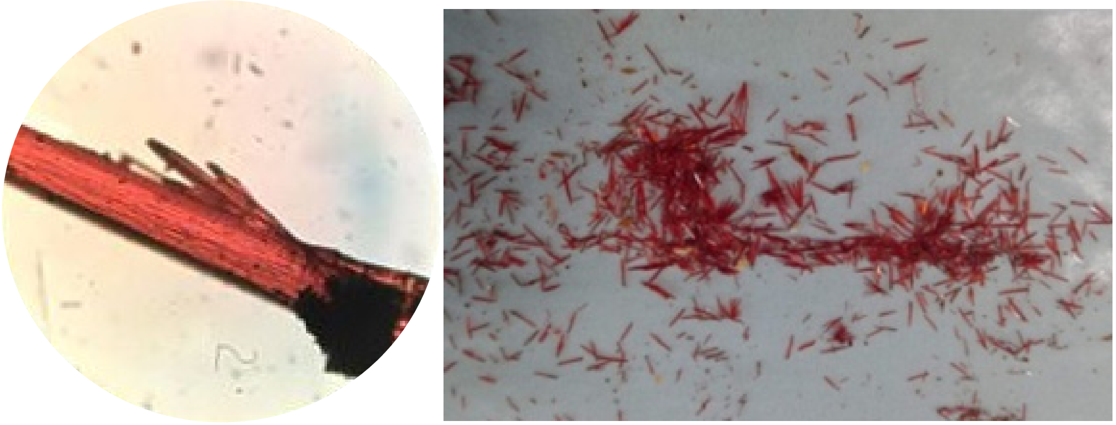

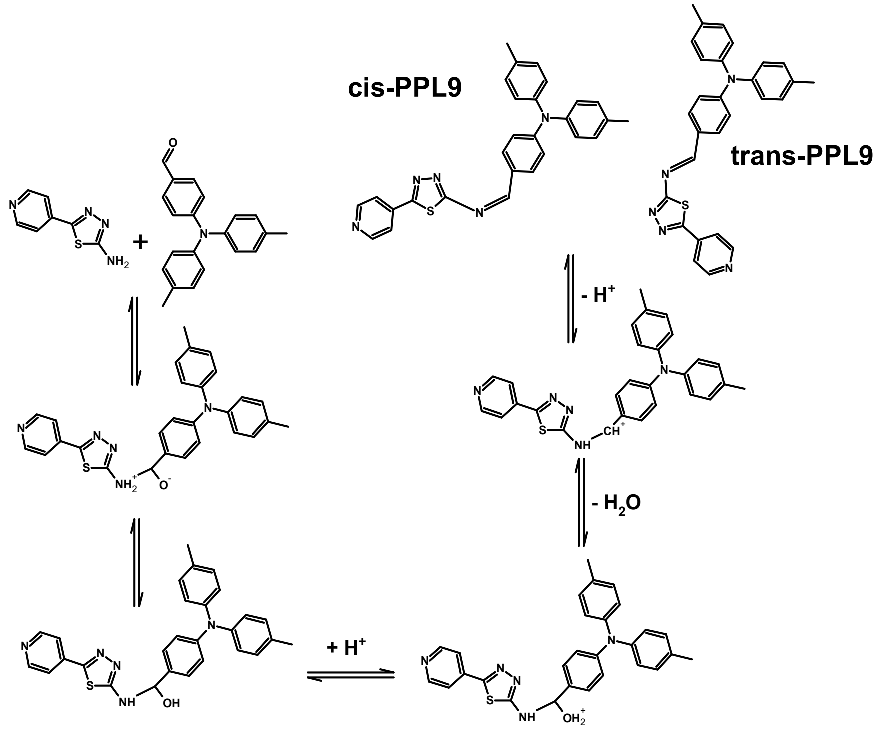

Figure S1. Photos of obtained crystals PPL9 and scheme of imine formation: aminocarbinol formation (left) and imine formation (right).

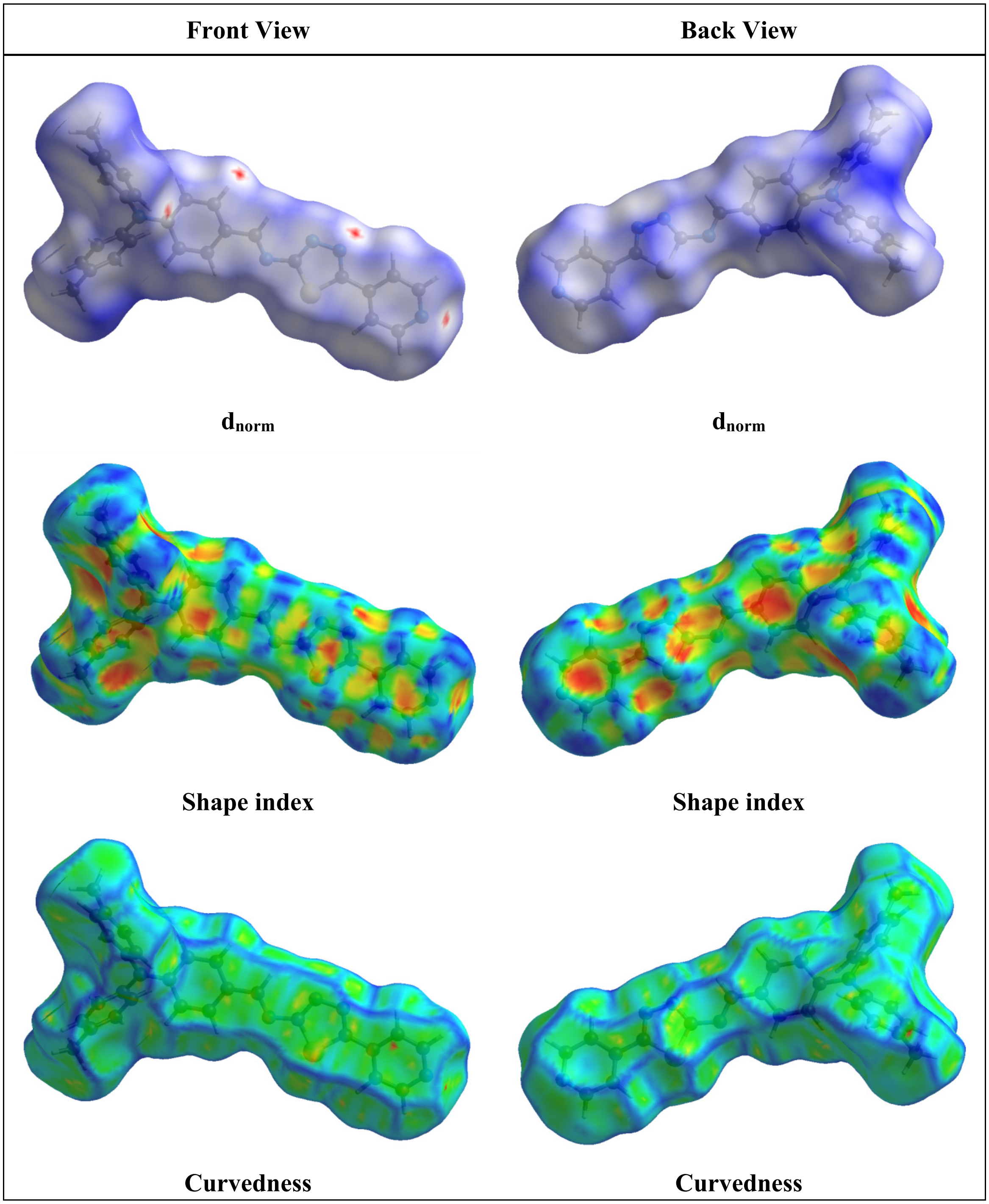

Figure S2. View front (left) and back (right) of the three-dimensional Hirshfeld surface present views of d_{norm}, shape index and curvedness surfaces of PPL9.

Table S1. The bond lengths (Å) and bond angles (°), with s.u.s in parentheses, determined for PPL9, by X-ray diffraction and the corresponding theoretical parameters, calculated for *trans*-PPL9 by B3LYP method 6-31+G (d,p) basis set.

	Lengths / Angles	Experimental for PPL9	Calculated for *trans*-PPL9
1	C1-C2	1.520(4)	1.510
2	C2-C3	1.363(4)	1.401
3	C3-C4	1.382(4)	1.393
4	C4-C5	1.372(3)	1.400
5	C5-C6	1.379(3)	1.402
6	C6-C7	1.379(4)	1.392
7	C7-C2	1.366(4)	1.402
8	C5-N1	1.428(3)	1.430
9	N1-C12	1.423(3)	1.430
10	C12-C13	1.376(3)	1.401
11	C13-C14	1.379(3)	1.393
12	C14-C9	1.376(3)	1.401
13	C9-C8	1.511(3)	1.510
14	C9-C10	1.375(3)	1.401
15	C10-C11	1.376(3)	1.393
16	C11-C12	1.384(3)	1.402
17	N1-C15	1.405(3)	1.400
18	C15-C16	1.393(3)	1.412
19	C16-C17	1.372(3)	1.385
20	C17-C18	1.392(3)	1.408
21	C18-C19	1.391(3)	1.411
22	C19-C20	1.370(3)	1.381
23	C20-C15	1.394(3)	1.416
24	C18-C21	1.457(3)	1.446
25	C21-N2	1.278(3)	1.298
26	N2-C22	1.385(3)	1.371
27	C22-N3	1.293(3)	1.317

28	N3-N4	1.371(3)	1.352
29	N4-C23	1.285(3)	1.313
30	C23-S1	1.712(2)	1.756
31	S1-C22	1.722(2)	1.763
32	C23-C24	1.474(3)	1.467
33	C24-C25	1.373(3)	1.402
34	C25-C26	1.382(3)	1.394
35	C26-N5	1.325(3)	1.338
36	N5-C27	1.326(4)	1.342
37	C27-C28	1.372(4)	1.391
38	C28-C24	1.385(3)	1.404
39	C1-C2-C3	121.5(3)	121.27
40	C2-C3-C4	122.4(3)	121.45
41	C3-C4-C5	119.9(3)	120.22
42	C4-C5-C6	118.3(2)	118.96
43	C5-C6-C7	120.3(3)	120.20
44	C6-C7-C2	121.8(3)	121.44
45	C7-C2-C1	121.4(3)	121.01
46	C7-C2-C3	117.1(3)	117.72
47	C4-C5-N1	121.3(2)	120.30
48	N1-C5-C6	120.3(2)	120.72
49	C5-N1-C15	119.14(18)	120.95
50	C12-N1-C15	120.24(18)	121.06
51	C12-N1-C5	120.10(17)	117.98
52	N1-C12-C13	121.3(2)	120.21
53	C12-C13-C14	120.7(2)	120.23
54	C13-C14-C9	121.6(2)	121.44
55	C14-C9-C10	117.3(2)	117.72
56	C14-C9-C8	121.5(2)	121.13
57	C8-C9-C10	121.3(2)	121.14

58	C9-C10-C11	121.8(2)	121.46
59	C10-C11-C12	120.5(2)	120.19
60	C11-C12-C13	118.1(2)	118.95
61	C11-C12-N1	120.6(2)	120.83
62	C20-C15-N1	121.5(2)	120.73
63	C16-C15-N1	120.3(2)	120.92
64	C15-C20-C19	120.8(2)	120.75
65	C20-C19-C18	120.9(2)	121.09
66	C19-C18-C17	118.4(2)	117.98
67	C18-C17-C16	120.7(2)	121.45
68	C17-C16-C15	121.09(2)	120.39
69	C16-C15-C20	118.2(2)	118.35
70	C17-C18-C21	120.0(2)	119.81
71	C19-C18-C21	121.7(2)	122.22
72	C18-C21-N2	121.7(2)	122.35
73	C21-N2-C22	117.7(2)	118.39
74	N2-C22-S1	118.93(17)	118.87
75	N2-C22-N3	127.6(2)	128.07
76	N3-C22-S1	113.42(17)	113.04
77	C22-N3-N4	112.0(2)	113.32
78	N3-N4-C23	113.9(2)	114.15
79	N4-C23-S1	113.05(17)	112.99
80	C23-S1-C22	87.60(11)	86.49
81	S1-C23-C24	124.70(18)	124.27
82	N4-C23-C24	122.3(2)	122.74
83	C23-C24-C25	122.4(2)	122.60
84	C23-C24-C28	120.1(2)	119.97
85	C25-C24-C28	117.5(2)	117.43
86	C24-C25-C26	119.1(3)	118.96
87	C25-C26-N5	124.2(3)	124.03

88	C26-N5-C27	115.8(2)	116.60
89	N5-C27-C28	124.6(3)	124.21
90	C27-C28-C24	118.8(3)	118.77

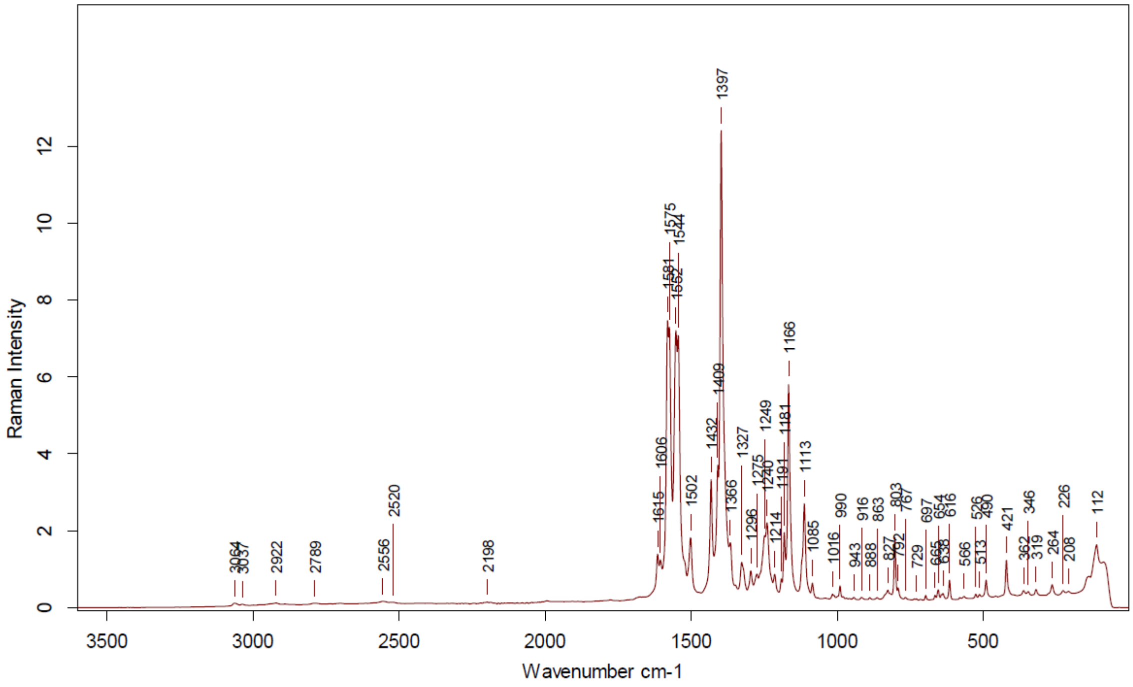

Figure S3. Experimental FT-Raman spectra of crystalline PPL9 range 3600–400 cm^{-1}.

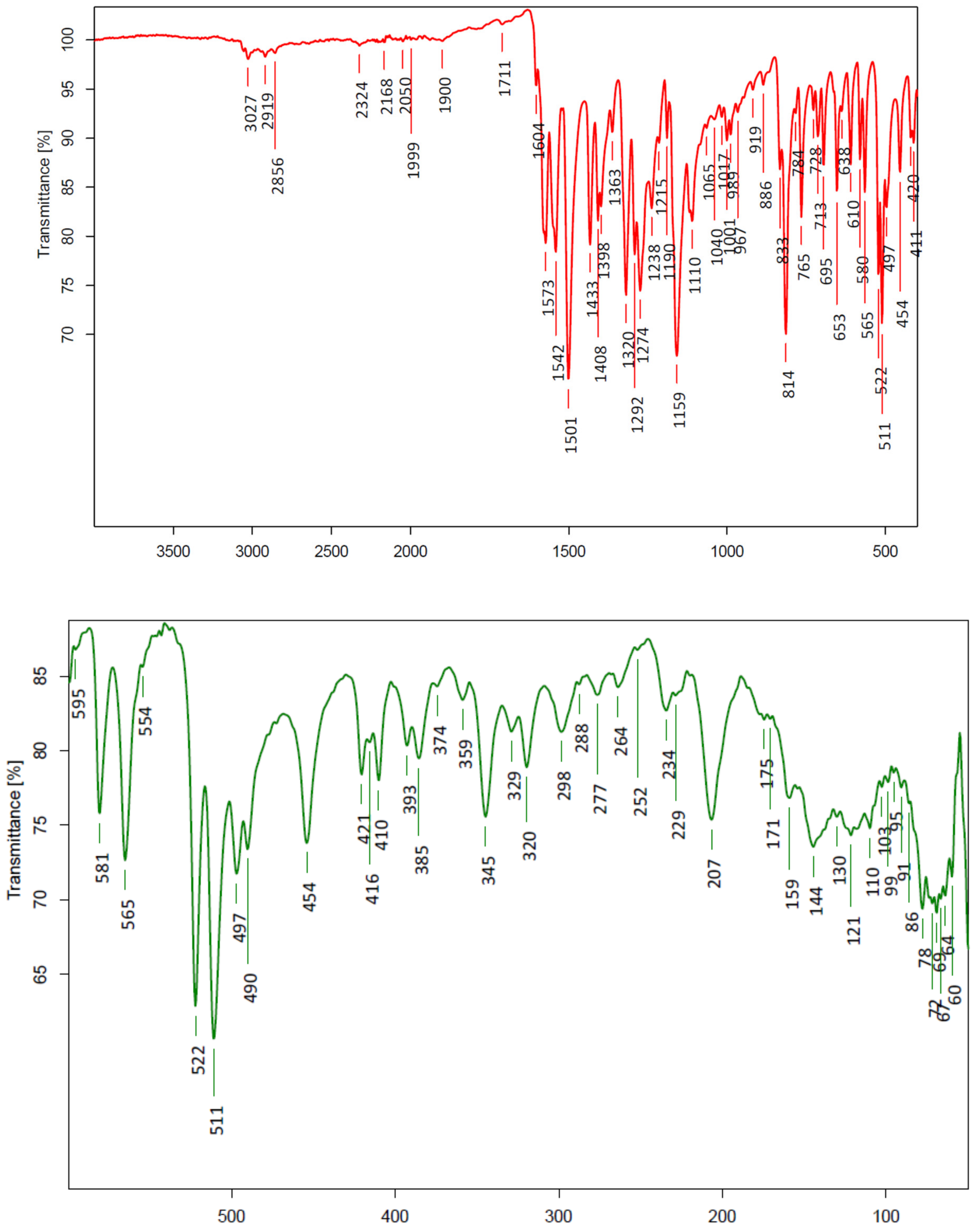

Figure S4. Experimental FT-IR spectra of crystalline PPL9 range 4000–400 cm^{-1} (red) and range 600–50 cm^{-1} (green).

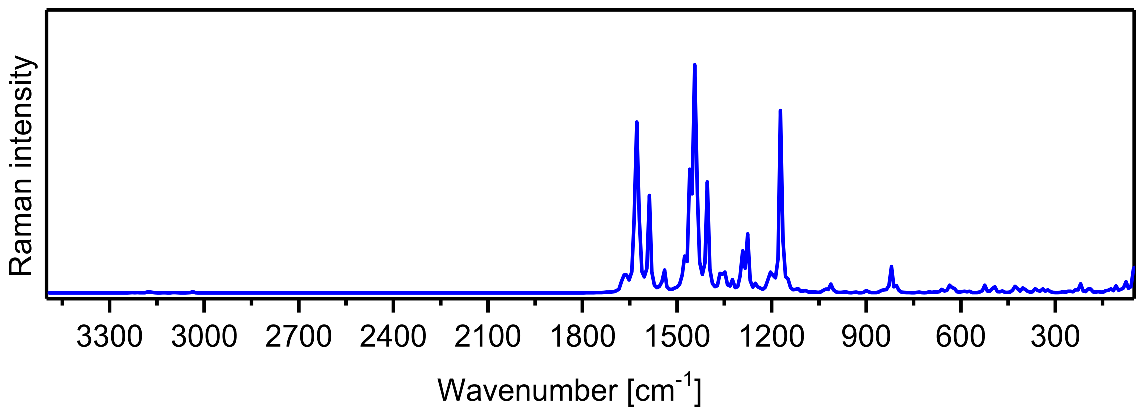

Figure S5. Theoretical scaled Raman spectra of PPL9 in the range 3500–50 cm^{-1}.

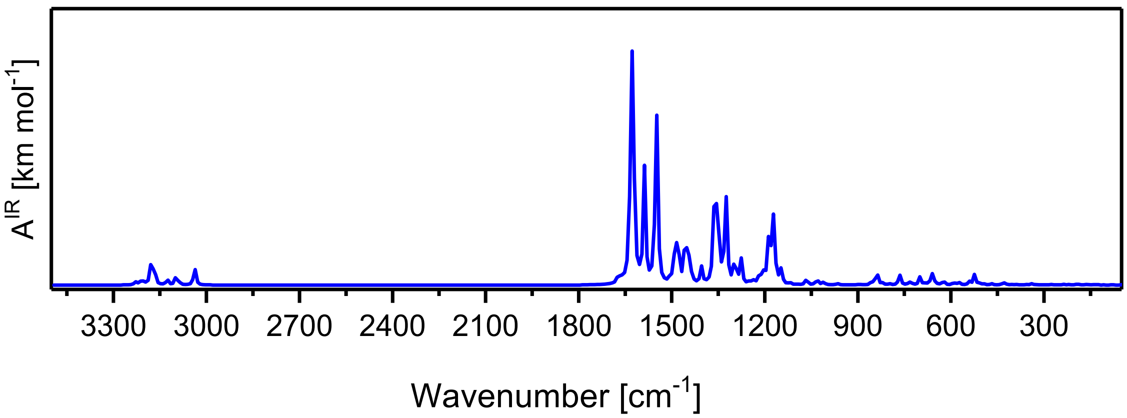

Figure S6. Theoretical scaled FT-IR spectra of PPL9 in the range in the range 3500–50 cm^{-1}.

Table S2. A statement of the theoretical harmonic wavenumbers (ν^a, ν^b, cm^{-1}), infrared intensities (A^{IR}, km mol cm^{-1}), Raman scattering activities (S^R, A^4 amu cm^{-1}) and Raman intensities (I^R) calculated for PPL9 by the B3LYP method with 6-31+G (d,p) basis set.

ν^a	A^{IR}	S^R	I^R	PED (%) Calculated by using the FCART 07 Program
3233	4	63	28	ν(H57C56)(98), ν(H55C54)(1)
3226	9	138	62	ν(H44C43)(62), ν(H42C41)(32), ν(H37C36)(4)
3225	8	84	38	ν(H37C36)(91), ν(H44C43)(3), ν(H39C38)(2)
3212	2	50	23	ν(H42C41)(65), ν(H44C43)(33)
3210	3	114	52	ν(H32C31)(82), ν(H29C28)(8), ν(H34C33)(7), ν(H27C26)(1)
3209	4	113	52	ν(H15C14)(82), ν(H13C12)(7), ν(H18C17)(5), ν(H29C28)(3)
3208	9	11	5	ν(H29C28)(79), ν(H32C31)(9), ν(H27C26)(6), ν(H15C14)(3)
3207	8	17	8	ν(H18C17)(86), ν(H20C19)(7), ν(H15C14)(5)
3199	7	76	35	ν(H51C50)(92), ν(H53C52)(7)
3182	8	91	43	ν(H39C38)(96), ν(H37C36)(3)
3180	33	121	57	ν(H34C33)(74), ν(H13C12)(11), ν(H32C31)(6), ν(H27C26)(4)
3179	21	121	57	ν(H13C12)(77), ν(H34C33)(12), ν(H15C14)(6), ν(H32C31)(1)
3179	15	116	55	ν(H27C26)(87), ν(H29C28)(6), ν(H34C33)(4)
3178	18	112	53	ν(H20C19)(90), ν(H18C17)(7), ν(H13C12)(1)
3170	49	402	191	ν(H55C54)(85), ν(H53C52)(11), ν(H51C50)(1), ν(H57C56)(1)
3166	35	230	109	ν(H53C52)(81), ν(H55C54)(12), ν(H51C50)(5)
3127	14	86	43	ν(H9C7)(62), ν(H8C7)(37)
3126	14	88	44	ν(H22C21)(52), ν(H23C21)(47)
3098	21	121	62	ν(H8C7)(41), ν(H10C7)(39), ν(H9C7)(19)
3098	20	94	48	ν(H24C21)(38), ν(H23C21)(32), ν(H22C21)(28)
3088	13	231	119	ν(H46C45)(99)
3038	40	460	248	ν(H10C7)(59), ν(H8C7)(21), ν(H9C7)(18)
3037	46	329	177	ν(H24C21)(60), ν(H23C21)(19), ν(H22C21)(18)
1674	28	892	1898	ν(C43C41)(8), ν(C38C36)(8), ν(C33C31)(5), ν(C28C26)(5)
1664	18	1333	2865	ν(C19C17)(11), ν(C14C12)(10), ν(C33C31)(9), ν(C28C26)(9)
1663	13	792	1704	ν(C45N3)(11), ν(C38C36)(10), ν(C43C41)(9), ν(C33C31)(5)
1648	11	392	857	ν(C56C54)(18), ν(C52C50)(18), ν(C50C49)(8), ν(C56C49)(5)
1631	894	14227	31765	ν(C45N3)(19), ν(C40C38)(9), δ(C40C45H46)(6), ν(C12C11)(5)
1627	431	3918	8787	ν(C31C30)(11), ν(C26C25)(10), ν(C30C28)(10), ν(C33C25)(9)
1621	297	3704	8357	ν(C19C11)(9), ν(C16C14)(9), ν(C12C11)(8), ν(C17C16)(8)
1606	16	297	681	ν(C50C49)(22), ν(C56C49)(20), ν(C52N6)(16), ν(C54N6)(10)
1588	536	7591	17787	ν(C45N3)(14), ν(C41C40)(14), ν(C36C35)(11), ν(C43C35)(9)
1556	2	28	69	δ(H32C31C33)(3), δ(H18C17C19)(3), δ(C17C19H20)(3), δ(C31C33H34)(3)

ν^a	A^{IR}	S^R	I^R	PED (%) Calculated by using the FCART 07 Program
1552	159	163	397	δ(C26C28H29)(6), δ(H32C31C33)(6), δ(H27C26C28)(5), δ(C31C33H34)(5)
1549	758	251	615	δ(H37C36C38)(7), δ(C41C43H44)(7), δ(C35C43H44)(6), δ(C36C38H39)(4)
1540	24	1514	3748	δ(H55C54C56)(15), δ(C50C52H53)(10), δ(C54C56H57)(8), ν(C54N6)(8)
1508	2	35	91	δ(H8C7H10)(26), δ(H9C7H10)(17), δ(H22C21H24)(12), δ(H23C21H24)(11)
1507	11	34	87	δ(H22C21H24)(23), δ(H23C21H24)(23), δ(H8C7H10)(14), δ(H9C7H10)(9)
1502	5	32	83	δ(H8C7H9)(65), δ(H9C7H10)(22), δ(H8C7C11)(4), δ(H8C7H10)(3)
1502	5	29	74	δ(H22C21H23)(68), δ(H23C21H24)(11), δ(H22C21H24)(10), δ(H22C21C25)(2)
1488	275	306	805	ν(C48N5)(22), ν(C47N3)(6), ν(C38C36)(5), ν(C49C48)(5)
1478	112	2067	5504	δ(C35C36H37)(11), ν(C43C41)(11), ν(C38C36)(9), ν(C48N5)(8)
1459	50	6122	16679	δ(H15C14C16)(5), ν(C14C12)(5), δ(C30C31H32)(5), δ(H9C7H10)(4)
1455	226	4148	11346	δ(N6C52H53)(17), δ(N6C54H55)(12), ν(C56C54)(7), δ(C49C56H57)(6)
1451	2	16	43	ν(C28C26)(6), ν(C33C31)(5), δ(H29C28C30)(5), δ(C25C33H34)(5)
1442	135	18389	51120	ν(C47N3)(14), ν(C48N5)(12), δ(N3C45H46)(11), ν(C45N3)(8)
1428	3	88	250	δ(H8C7H10)(22), δ(H9C7H10)(17), δ(H10C7C11)(14), δ(H8C7C11)(12)
1428	1	89	252	δ(H23C21H24)(20), δ(H22C21H24)(19), δ(H24C21C25)(14), δ(H22C21C25)(12)
1404	78	6689	19451	ν(C47N4)(29), δ(N3C45H46)(19), δ(C40C45H46)(9), ν(C45N3)(9)
1368	20	70	212	δ(H55C54C56)(14), δ(C49C56H57)(13), δ(C49C50H51)(12), δ(C54C56H57)(8)
1362	61	316	968	ν(C36C35)(9), ν(C43C35)(6), ν(C30N2)(6), ν(C43C41)(5)
1360	615	1341	4118	ν(C35N2)(10), δ(C25C33H34)(6), δ(C31C33H34)(6), δ(C25C26H27)(6)
1347	155	937	2922	ν(C41C40)(11), δ(C41C43H44)(10), ν(C40C38)(9), δ(C40C41H42)(7)
1345	18	8	26	δ(C12C14H15)(5), δ(H15C14C16)(5), δ(C16C17H18)(5), δ(H18C17C19)(4)
1342	2	49	155	ν(C33C25)(8), ν(C26C25)(7), ν(C35N2)(7), ν(C12C11)(6)
1326	458	664	2126	ν(C35N2)(23), ν(C17C16)(10), ν(C16C14)(7), ν(C30C28)(4)
1318	14	10	33	ν(C30C28)(11), ν(C31C30)(11), ν(C16C14)(8), ν(C17C16)(6)
1301	7	59	196	ν(C49C48)(13), ν(C48N5)(12), ν(C52C50)(10), ν(C52N6)(8)
1297	101	594	1972	ν(C30N2)(26), ν(C16N2)(24), ν(C43C35)(4), ν(C36C35)(4)
1291	21	1878	6287	ν(C52N6)(17), ν(C50C49)(15), ν(C54N6)(13), ν(C56C54)(10)
1277	120	2969	10117	ν(C45C40)(17), δ(N3C45H46)(8), ν(C49C48)(6), ν(C43C41)(5)
1250	10	425	1499	δ(N6C52H53)(14), δ(N6C54H55)(14), ν(C54N6)(11), δ(H51C50C52)(8)
1239	11	94	335	ν(C11C7)(17), ν(C25C21)(11), δ(H13C12C14)(4), δ(C17C19H20)(4)
1238	9	40	141	ν(C25C21)(22), ν(C11C7)(17), δ(C26C25C33)(3), ν(C28C26)(3)
1221	29	52	190	ν(C14C12)(8), ν(C33C31)(8), ν(C28C26)(8), ν(C19C17)(8)
1207	61	868	3237	δ(N5N4C47)(7), ν(C47N3)(7), δ(C41C43H44)(6), δ(H37C36C38)(5)
1207	14	250	932	δ(C12C14H15)(5), δ(H18C17C19)(5), δ(H32C31C33)(5), δ(C26C28H29)(5)
1198	15	483	1824	ν(C30N2)(7), ν(C16N2)(7), ν(C17C16)(3), δ(C36C38H39)(3)
1185	326	153	589	δ(C36C38H39)(10), ν(C47N3)(10), δ(H37C36C38)(7), δ(H39C38C40)(6)

ν[a]	A[IR]	S[R]	I[R]	PED (%) Calculated by using the FCART 07 Program
1171	329	8682	33983	ν(N5N4)(58), δ(N4N5C48)(10), δ(N3C47N4)(4), δ(N5N4C47)(3)
1149	64	343	1381	δ(H42C41C43)(19), δ(C41C43H44)(13), δ(H37C36C38)(10), ν(C38C36)(7)
1146	2	2	8	δ(H32C31C33)(9), δ(H27C26C28)(8), δ(C31C33H34)(8), δ(C26C28H29)(7)
1144	15	30	123	δ(H18C17C19)(8), δ(C12C14H15)(8), δ(C17C19H20)(7), δ(H13C12C14)(7)
1119	9	185	777	δ(C54C56H57)(18), δ(H51C50C52)(16), ν(C52C50)(15), δ(C50C52H53)(10)
1095	3	96	417	δ(C49C56H57)(20), δ(C49C50H51)(15), δ(N6C54C56)(9), ν(C54N6)(8)
1065	16	13	58	δ(H10C7C11)(50), δ(H8C7C11)(16), δ(H9C7C11)(7), γ(H13(C12C11C14))(4)
1065	16	15	67	δ(H24C21C25)(51), δ(H23C21C25)(13), δ(H22C21C25)(10), γ(C21(C25C26C33))(4)
1037	4	15	71	δ(C11C12C14)(11), δ(C16C17C19)(11), δ(C11C19C17)(11), δ(C12C14C16)(11)
1036	5	36	168	δ(C25C26C28)(11), δ(C25C33C31)(11), δ(C30C31C33)(11), δ(C26C28C30)(11)
1029	17	98	469	γ(H46(C45N3C40))(76), γ(H39(C38C36C40))(4), δ(C52N6C54)(2), γ(C38(C40C41C45))(2)
1024	1	4	17	γ(H46(C45N3C40))(16), δ(C52N6C54)(14), δ(C50C49C56)(9), δ(N4N5C48)(6)
1014	3	230	1120	δ(C40C41C43)(12), δ(C36C38C40)(12), δ(C35C43C41)(11), δ(C35C36C38)(10)
1013	1	5	26	δ(H9C7C11)(36), δ(H8C7C11)(28), ν(C19C11)(7), ν(C12C11)(5)
1012	1	3	13	δ(H22C21C25)(34), δ(H23C21C25)(31), ν(C33C25)(6), ν(C26C25)(5)
1009	13	161	791	γ(H55(C54N6C56))(65), γ(H57(C56C49C54))(36), γ(H53(C52N6C50))(10), γ(H51(C50C49C52))(1)
1003	0.2	2	10	ν(C56C49)(15), ν(C54N6)(14), ν(C56C54)(12), ν(C52N6)(11)
992	1	1	7	γ(H42(C41C40C43))(71), γ(H44(C43C35C41))(41), γ(H46(C45N3C40))(2)
981	0.3	5	27	γ(H53(C52N6C50))(71), γ(H51(C50C49C52))(29), γ(H57(C56C49C54))(12), γ(H55(C54N6C56))(3)
970	0.5	11	56	γ(H20(C19C11C17))(25), γ(H13(C12C11C14))(23), γ(H18(C17C16C19))(21), γ(H15(C14C12C16))(19)
969	1	5	28	γ(H34(C33C25C31))(30), γ(H32(C31C30C33))(25), γ(H27(C26C25C28))(22), γ(H29(C28C26C30))(16)
967	4	25	128	γ(H15(C14C12C16))(29), γ(H13(C12C11C14))(21), γ(H39(C38C36C40))(13), γ(H18(C17C16C19))(12)
965	1	3	15	γ(H29(C28C26C30))(22), γ(H39(C38C36C40))(21), γ(H37(C36C35C38))(17), γ(H32(C31C30C33))(16)
963	2	1	6	γ(H39(C38C36C40))(22), γ(H37(C36C35C38))(15), γ(H18(C17C16C19))(15), γ(H20(C19C11C17))(12)
939	0.2	3	15	ν(C30C28)(9), ν(C17C16)(8), δ(C16N2C35)(5), ν(C31C30)(4)
935	1	31	170	ν(C43C35)(9), ν(C36C35)(7), δ(C16N2C30)(6), ν(C16C14)(5)
899	2	70	405	ν(C41C40)(17), δ(C45N3C47)(15), δ(N3C45C40)(12), δ(C40C45H46)(9)
891	2	4	22	γ(H57(C56C49C54))(46), γ(H51(C50C49C52))(27), γ(H55(C54N6C56))(25), γ(H53(C52N6C50))(1)

ν^a	A^{IR}	S^R	I^R	PED (%) Calculated by using the FCART 07 Program
856	18	13	79	γ(H44(C43C35C41))(45), γ(H42(C41C40C43))(22), γ(C38(C40C41C45))(4), γ(H37(C36C35C38))(4)
850	0.4	44	275	γ(H15(C14C12C16))(21), γ(H13(C12C11C14))(18), γ(H18(C17C16C19))(16), γ(H20(C19C11C17))(12)
846	2	18	116	γ(H32(C31C30C33))(18), γ(H29(C28C26C30))(16), γ(H34(C33C25C31))(13), γ(H27(C26C25C28))(11)
841	21	1	8	γ(H51(C50C49C52))(43), γ(H53(C52N6C50))(22), γ(H55(C54N6C56))(12), γ(H57(C56C49C54))(9)
839	4	41	260	γ(H37(C36C35C38))(32), γ(H39(C38C36C40))(22), γ(H13(C12C11C14))(9), γ(H15(C14C12C16))(9)
838	37	9	57	γ(H34(C33C25C31))(14), γ(H32(C31C30C33))(12), γ(H27(C26C25C28))(11), γ(H29(C28C26C30))(9)
833	4	4	27	γ(H37(C36C35C38))(13), γ(H18(C17C16C19))(11), γ(H20(C19C11C17))(10), γ(H29(C28C26C30))(8)
821	8	659	4346	ν(C48S1)(8), ν(C47N3)(8), δ(N5N4C47)(6), δ(N4N5C48)(6)
804	2	140	950	ν(C48S1)(8), ν(C25C21)(7), δ(N5N4C47)(6), ν(C11C7)(6)
796	4	15	103	ν(C11C7)(14), ν(C25C21)(14), δ(C14C16C17)(9), δ(C28C30C31)(9)
765	50	1	11	ν(C47S1)(25), δ(N4N5C48)(6), ν(C45C40)(6), δ(N5N4C47)(6)
754	0	1	4	τ(N6C52C50C49)(31), τ(C49C56C54N6)(31), γ(C48(C49C50C56))(25), τ(C50C52N6C54)(10)
736	6	14	110	γ(C36(C35N2C43))(27), τ(C35C43C41C40)(25), τ(C40C38C36C35)(24), γ(C38(C40C41C45))(8)
729	13	11	83	τ(C11C19C17C16)(16), τ(C16C14C12C11)(16), γ(C14(C16N2C17))(12), τ(C30C28C26C25)(11)
725	2	1	9	τ(C25C33C31C30)(18), τ(C30C28C26C25)(18), τ(C16C14C12C11)(14), τ(C11C19C17C16)(13)
700	37	18	151	δ(C52N6C54)(20), ν(C48S1)(19), ν(C49C48)(8), ν(C50C49)(7)
681	8	17	142	δ(N6C54C56)(17), δ(C49C50C52)(15), δ(N6C52C50)(12), δ(C49C56C54)(11)
662	61	62	544	ν(C47S1)(8), δ(C38C40C41)(6), δ(C41C40C45)(5), δ(S1C48N5)(4)
659	0.5	5	47	δ(C11C12C14)(8), δ(C11C19C17)(7), δ(C16C17C19)(6), δ(C12C14C16)(6)
652	8	8	73	δ(C25C26C28)(7), δ(C30C31C33)(6), δ(C26C28C30)(6), δ(C25C33C31)(5)
647	4	11	100	δ(C36C38C40)(13), δ(C35C43C41)(11), δ(C40C41C43)(8), δ(C35C36C38)(8)
637	1	113	1050	τ(S1C47N4N5)(18), τ(C48N5N4C47)(17), γ(N3(C47S1N4))(15), τ(C40C45N3C47)(5)
624	14	120	1142	δ(C47S1C48)(8), δ(S1C47N4)(6), τ(S1C47N4N5)(6), τ(C48N5N4C47)(6)
622	9	6	56	γ(C49(C48S1N5))(29), τ(S1C48N5N4)(13), τ(N5C48S1C47)(11), γ(C48(C49C50C56))(7)
592	12	33	332	δ(C16N2C30)(7), γ(C7(C11C12C19))(6), δ(C26C25C33)(5), γ(C21(C25C26C33))(5)
575	17	30	313	δ(C12C11C19)(8), ν(C11C7)(7), δ(C30N2C35)(6), δ(C26C25C33)(6)

ν^a	A^{IR}	S^R	I^R	PED (%) Calculated by using the FCART 07 Program
540	15	4	48	γ(C36(C35N2C43))(15), γ(C14(C16N2C17))(8), τ(C43C41C40C38)(7), τ(C41C40C38C36)(6)
526	8	66	781	γ(C7(C11C12C19))(13), γ(C14(C16N2C17))(10), δ(N3C45C40)(5), δ(N3C47N4)(5)
524	42	36	425	γ(C48(C49C50C56))(13), γ(C28(C30N2C31))(11), τ(C50C52N6C54)(10), τ(C56C54N6C52)(10)
509	6	6	78	γ(C7(C11C12C19))(12), γ(C14(C16N2C17))(11), γ(C36(C35N2C43))(9), γ(C21(C25C26C33))(5)
495	7	112	1419	γ(C21(C25C26C33))(12), γ(C28(C30N2C31))(9), ν(C47S1)(8), δ(C38C40C45)(6)
470	7	22	301	δ(N2C30C31)(10), δ(N2C16C14)(8), δ(N2C30C28)(8), δ(N2C16C17)(7)
431	12	44	662	τ(C40C38C36C35)(31), τ(C35C43C41C40)(27), τ(C41C40C38C36)(12), τ(C38C36C35N2)(9)
425	2	47	720	τ(C25C33C31C30)(26), τ(C30C28C26C25)(25), τ(C16C14C12C11)(7), τ(C11C19C17C16)(7)
423	3	17	263	τ(C11C19C17C16)(27), τ(C16C14C12C11)(26), τ(C30C28C26C25)(10), τ(C25C33C31C30)(9)
402	2	51	837	δ(C48C49C50)(14), δ(N5C48C49)(12), ν(C48S1)(9), δ(S1C48C49)(8)
395	1	12	199	δ(C21C25C33)(7), δ(C21C25C26)(7), δ(C7C11C12)(7), δ(C7C11C19)(6)
389	2	4	73	τ(N6C52C50C49)(18), τ(C56C54N6C52)(13), γ(C49(C48S1N5))(8), τ(C40C45N3C47)(8)
384	1	2	30	τ(C49C56C54N6)(31), τ(N6C52C50C49)(19), τ(C50C52N6C54)(14), τ(C54C56C49C48)(10)
364	0.4	30	543	δ(C7C11C12)(7), δ(C7C11C19)(7), ν(C49C48)(4), γ(C21(C25C26C33))(4)
356	2	10	180	δ(C21C25C26)(14), δ(C21C25C33)(10), δ(C7C11C19)(8), δ(C7C11C12)(4)
342	3	21	405	γ(C38(C40C41C45))(22), τ(C41C43C35N2)(7), τ(C35C43C41C40)(6), τ(N3C47S1C48)(6)
337	5	15	298	γ(C21(C25C26C33))(8), τ(C33C31C30N2)(8), τ(N2C30C28C26)(8), τ(C31C33C25C21)(7)
322	2	24	501	δ(N2C35C36)(11), δ(N2C35C43)(11), τ(C19C17C16N2)(4), τ(N2C16C14C12)(4)
296	2	2	53	δ(C21C25C33)(7), δ(C7C11C12)(7), δ(N2C16C14)(5), δ(C21C25C26)(5)
277	2	6	161	ν(C49C48)(10), ν(C35N2)(6), δ(N2C16C14)(3), δ(N2C30C31)(3)
256	2	13	351	δ(N2C16C17)(11), δ(N2C30C28)(10), ν(C16N2)(7), δ(C7C11C19)(6)
237	4	11	340	δ(S1C47N3)(7), δ(C38C40C45)(7), δ(N3C47N4)(5), δ(C41C40C45)(5)
221	2	42	1343	τ(N3C45C40C38)(16), τ(C52C50C49C48)(7), τ(C54C56C49C48)(7), τ(N3C47S1C48)(7)
192	7	26	977	τ(N3C45C40C38)(27), τ(C38C36C35N2)(15), τ(C41C43C35N2)(10), τ(C40C45N3C47)(6)
165	2	3	122	τ(C31C33C25C21)(12), τ(C28C26C25C21)(12), τ(C17C19C11C7)(9), τ(C14C12C11C7)(9)

ν^a	A^{IR}	S^R	I^R	PED (%) Calculated by using the FCART 07 Program
155	1	0.2	9	τ(C28C26C25C21)(9), τ(C17C19C11C7)(8), τ(C31C33C25C21)(8), τ(C14C12C11C7)(8)
137	2	7	374	τ(C36C35N2C16)(6), τ(C52C50C49C48)(6), τ(C54C56C49C48)(5), τ(C17C19C11C7)(5)
125	2	8	472	δ(C45N3C47)(8), τ(C52C50C49C48)(6), τ(N3C45C40C38)(6), τ(C54C56C49C48)(6)
107	1	13	889	δ(S1C48C49)(11), δ(N5C48C49)(11), δ(C41C40C45)(9), δ(C48C49C56)(8)
88	1	6	544	τ(C36C35N2C16)(24), γ(C49(C48S1N5))(12), τ(N3C45C40C38)(7), γ(C38(C40C41C45))(5)
76	0.5	13	1289	γ(C16(N2C30C35))(20), τ(C14C16N2C30)(17), τ(C36C35N2C16)(15), τ(C28C30N2C16)(11)
50	0.3	11	1648	τ(C28C30N2C16)(28), τ(N2C16C14C12)(7), τ(C19C17C16N2)(6), τ(C50C49C48S1)(5)
48	0.5	19	3054	τ(C14C16N2C30)(13), τ(C33C31C30N2)(13), τ(N2C30C28C26)(13), γ(C16(N2C30C35))(12)
45	0.3	1	166	τ(C12C11C7H8)(73), τ(C50C49C48S1)(7), δ(C16N2C30)(2), δ(C30N2C35)(1)
42	1	1	223	τ(C50C49C48S1)(48), τ(C12C11C7H8)(14), γ(C16(N2C30C35))(11), τ(C14C16N2C30)(5)
39	1	3	583	τ(C28C30N2C16)(23), τ(C14C16N2C30)(21), γ(C16(N2C30C35))(10), τ(C50C49C48S1)(5)
37	2	6	1297	τ(C50C49C48S1)(34), τ(C14C16N2C30)(30), δ(C30N2C35)(4), δ(C16N2C30)(4)
34	0.4	1	120	τ(C26C25C21H22)(96)
33	2	10	2258	τ(C28C30N2C16)(27), τ(C36C35N2C16)(27), δ(C16N2C35)(6), τ(S1C47N3C45)(4)
21	0.2	5	1984	δ(C45N3C47)(11), τ(C36C35N2C16)(9), τ(S1C47N3C45)(7), δ(N3C45C40)(7)
18	1	7	3155	τ(S1C47N3C45)(39), τ(C40C45N3C47)(23), γ(C16(N2C30C35))(15), γ(C38(C40C41C45))(6)
14	0.2	5	2891	τ(S1C47N3C45)(28), τ(N3C45C40C38)(10), γ(C38(C40C41C45))(9), τ(C40C45N3C47)(8)

Abbreviations: ν, stretching; δ, in-plane bending; γ, out-of-plane bending; τ, torsion;

[a]The calculated B3LYP frequencies were non scaled,

[b]The calculated B3LYP frequencies were scaled: ν NH, and ν CH by 0.96 and the remaining modes by 0.98.

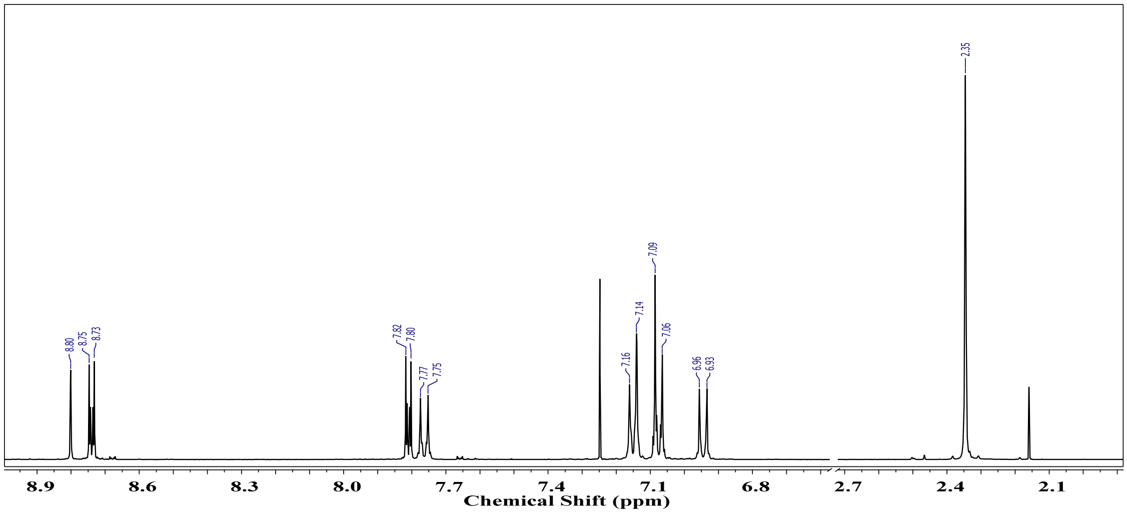

Figure S7. Experimental ^{1}H NMR spectra of crystalline PPL9.

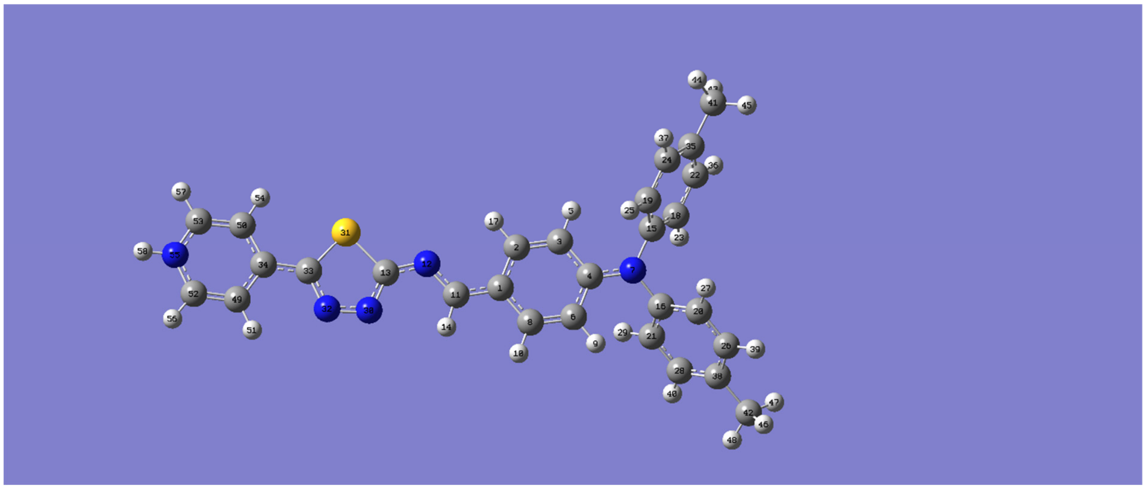

(a)

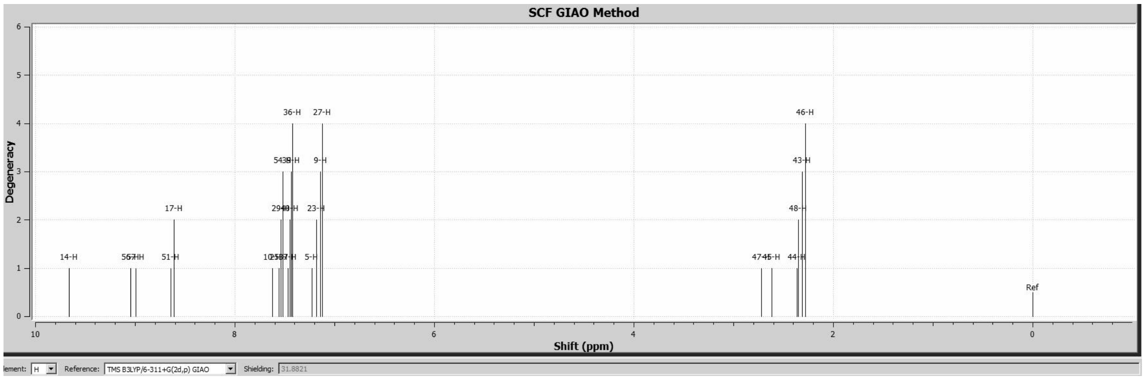

(b)

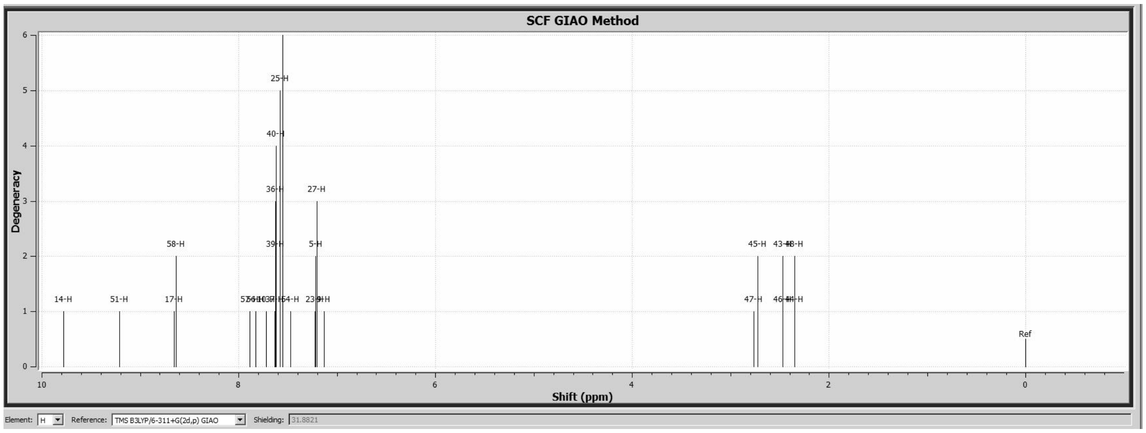

(c)

Figure S8. Simulated ^{1}H NMR spectrum of *trans*-PPL9 according to GIAO method[i] at DFT/B3LYP/6-311+G(2d,p) level. Numbering of *trans*-PPL9 (H^+ doped) (**a**) ^{1}H NMR spectrum of *trans*-PPL9 (**b**) ^{1}H NMR spectrum of H^+ doped *trans*-PPL9 (**c**).

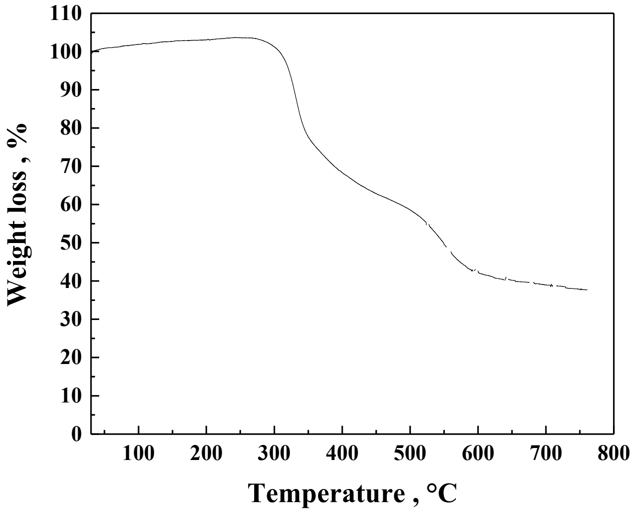

Figure S9. TGA of PPL9.

Reference

[i] K. Wolinski, J. F. Hilton, and P. Pulay, Efficient Implementation of the Gauge-Independent Atomic Orbital Method for NMR Chemical Shift Calculations, *J. Am. Chem. Soc.*, **1990**, *112*, 8251–60.
